# Context‐Dependent Role of GDF15: GDF15^+^ Tumor‐Associated Macrophages Suppress OSCC Progression by Enhancing Phagocytosis

**DOI:** 10.1002/advs.202518525

**Published:** 2026-03-13

**Authors:** Xinyu Zhou, Zhihang Zhou, Houyu Ju, Chaoyang Guo, Fangxing Zhu, Yiyi Zhang, Xu Chen, Tongchao Zhao, Laiping Zhong, Jingzhou Hu

**Affiliations:** ^1^ Department of Oral and Maxillofacial‐Head and Neck Oncology Ninth People's Hospital College of Stomatology Shanghai Jiao Tong University School of Medicine Shanghai P. R. China; ^2^ Shanghai Key Laboratory of Stomatology & Shanghai Research Institute of Stomatology & National Center for Stomatology National Clinical Research Center for Oral Diseases Shanghai P. R. China; ^3^ Department of Stomatology Huashan Hospital Fudan University Shanghai P. R. China; ^4^ Department of Oral Pathology Ninth People's Hospital College of Stomatology Shanghai Jiao Tong University School of Medicine Shanghai P. R. China; ^5^ Department of Laboratory Medicine Shanghai Ninth People's Hospital Shanghai Jiao Tong University School of Medicine Shanghai P. R. China; ^6^ Faculty of Medical Laboratory Science College of Health Science and Technology Shanghai Jiao Tong University School of Medicine Shanghai P. R. China

**Keywords:** growth differentiation factor 15, immune checkpoint blockade, oral squamous cell carcinoma, phagocytosis

## Abstract

The growth differentiation factor 15 (GDF15) neutralizing antibody has shown to overcome resistance to immune checkpoint blockade (ICB) in various solid tumors. Though the therapeutic effect of GDF15 neutralizing antibody indicates a protumor role of secreted GDF15, the double‐edged effect of GDF15 has remained a mystery for a long. Herein, we performed single‐cell RNA‐sequencing on oral squamous cell carcinoma (OSCC) samples before and after ICB‐based therapy to explore the context‐dependent functions of GDF15 across distinct cellular subsets. It revealed that GDF15^+^ macrophages were enriched in ICB‐sensitive OSCCs after treatment and might participate in mediating tumor regression. *Gdf15* ^fl/fl^ *Lyz2* ^Cre^ mice illustrated that GDF15 deficiency in macrophages could accelerate tumor progression by suppressing the infiltration of CD8^+^ T cells. Mechanistically, macrophage‐intrinsic GDF15 could enhance the abilities to phagocyte tumor cells and to cross‐present antigens to CD8^+^ T cells. The functional enhancement of GDF15 was mediated through the upregulation of the NF‐κB signaling pathway in macrophages. Collectively, the cellular source of GDF15 could determine its effect on tumor progression, with GDF15^+^ macrophages exerting an antitumor role in OSCC, whereas secreted GDF15 exerting a protumor role. The latter could be neutralized by the GDF15 antibody. These findings would advance a comprehensive understanding of the double‐edged effect of GDF15.

## Graphical abstract

This study identifies GDF15^+^ TAMs as a cell subset mediating tumor regression after immunotherapy. Macrophage‐intrinsic GDF15 enhances phagocytosis and antigen cross‐presentation to CD8^+^ T cells through the NF‐κB signaling pathway, thereby inhibiting tumor progression. We propose that GDF15 neutralization could exert its therapeutic effect on ablating the protumor effect of secreted GDF15 while sparing the antitumor effect of macrophage‐intrinsic GDF15.

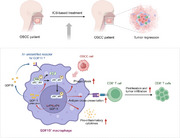

## 1 Introduction

In recent years, immune checkpoint blockade (ICB) therapy has shown promising clinical potential in the treatment of locally advanced oral squamous cell carcinoma (OSCC) [1]. The combination of neoadjuvant and adjuvant programmed cell death 1 (PD‐1) antibody monotherapy has increased the 3‐year event‐free survival rate from 46.4% to 57.6% in patients with locally advanced head and neck squamous‐cell carcinoma (HNSCC) in KEYNOTE‐689 [1]. Combination therapies of PD‐1 antibody with chemotherapy or targeted therapy have also been extensively investigated in locally advanced OSCC, showing favorable major pathological response (MPR) rates of approximately 40%–69% in neoadjuvant therapy [2, 3, 4, 5, 6]. Moreover, it has been validated that those who achieved MPR could possess significantly prolonged survival compared with non‐MPR patients [7], which is of great importance for improving the prognosis of OSCC.

However, there is still a subset of patients who have poor responses to ICB‐based treatment, which currently remains a major challenge for the therapy. Various drugs have been attempted to be incorporated into the ICB‐based therapy strategies to improve treatment response, including targeted drugs such as apatinib [2] and chemotherapeutics such as paclitaxel and cisplatin [3]. It was recently reported that growth differentiation factor 15 (GDF15) neutralizing antibody visugromab could overcome anti‐PD‐1 resistance in solid tumors, increasing objective response rates (ORRs) in patients who were relapsed or refractory to prior anti‐PD‐1 or anti‐programmed cell death ligand 1 (PD‐L1) treatment and providing retreatment attempts [8]. This finding presented an important opportunity for improving the application of ICB.

GDF15, also known as macrophage inhibitory cytokine 1 (MIC‐1), is a divergent member of the transforming growth factor β (TGF‐β) superfamily and was first identified in macrophages, where it functioned to limit their excessive activation under specific stimulations [9, 10, 11]. Subsequent studies have revealed that GDF15 is a stress‐responsive factor, which could be widely expressed under various pathological conditions and involved in the regulation of multiple diseases, including cancers [12]. However, GDF15 was considered as a “double‐edged sword” in cancers for years. Under most circumstances, GDF15 was reported to be overexpressed in various solid tumors compared with adjacent normal tissues [13, 14]. Patients with high tumor expression and high serum concentration of GDF15 tended to exhibit worse prognosis [15, 16], while blocking secreted GDF15 by neutralizing antibody could exhibit therapeutic effect on tumors [8], indicating a protumor role of GDF15. Tumor cells with GDF15 overexpression were also proved to possess stronger proliferation, migration, and invasion capabilities in various types of cancers, including head and neck squamous cell carcinoma [14], ovarian cancer [17] and so on. However, GDF15 was reported to exert an antitumor role in some cases. Overexpression of GDF15 could result in smaller tumor volumes and longer survival times in transgenic adenocarcinoma of mouse prostate (TRAMP) mice that naturally develop prostate cancer [18], indicating a protective role of GDF15 in the early stage of prostate cancer development. The increased expression of GDF15 induced by nonsteroidal anti‐inflammatory drug (NSAIDs) could inhibit the occurrence and tumoral transformation of colon polyps [19] and promote the apoptosis of colon cancer cells [20], indicating the impact of pharmacological intervention on the effect of GDF15. Moreover, we could observe bidirectional regulatory effects of GDF15 in one single case. Zhong et al. [21, 22] reported that the locally advanced OSCC patients with high GDF15 expression exhibited a lower survival; however, these patients could significantly benefit from docetaxel, cisplatin, and fluorouracil (TPF) neoadjuvant therapy than those with low GDF15 expression, indicating the complex role of GDF15.

So far, the double‐edged regulatory effect of GDF15 is still confusing and is speculated to vary due to numerous factors, such as tumor development stages, therapeutic interventions, cancer types, cell subtypes within tumor microenvironment (TME), functioning form of GDF15, and so on. Regrettably, the mechanism responsible for the double‐edged effect of GDF15 has not yet been discovered, which may lead to an unpredictable impact on the therapeutic effect of GDF15‐neutralizing antibody and become one of the major obstacles to its clinical application in cancer therapy.

In this study, we aimed to explore the functions of GDF15 in different cell subtypes within TME utilizing the single‐cell RNA sequencing (scRNA‐seq) results of OSCC samples before and after ICB‐based treatment, and to identify the key subpopulation contributing to the antitumor function of GDF15, which is opposite to the therapeutic effect of GDF15 neutralizing antibody. We discovered that GDF15^+^ tumor‐associated macrophages (TAMs) were enriched in the post‐treatment OSCCs sensitive to ICB‐based therapy and could mediate tumor regression after treatment. *Gdf15* ^fl/fl^ *Lyz2* ^Cre^ mice were further used to demonstrate that GDF15 expression in macrophages could enhance phagocytosis and antigen cross‐presentation abilities through the NF‐κB signaling pathway, thereby inhibiting tumor progression.

We hypothesize that the cellular source of GDF15 could determine its effect on tumor progression, with GDF15^+^ macrophages exerting an antitumor role in OSCC by enhancing phagocytosis and CD8^+^T cell immunity, whereas secreted GDF15 exerting a protumor role. The net effect of GDF15 in the TME is dependent on the balance between the two effects.

## 2 Results

### 2.1 GDF15^+^ TAMs are Enriched in OSCCs Sensitive to ICB‐Based Therapy

**FIGURE 1:**
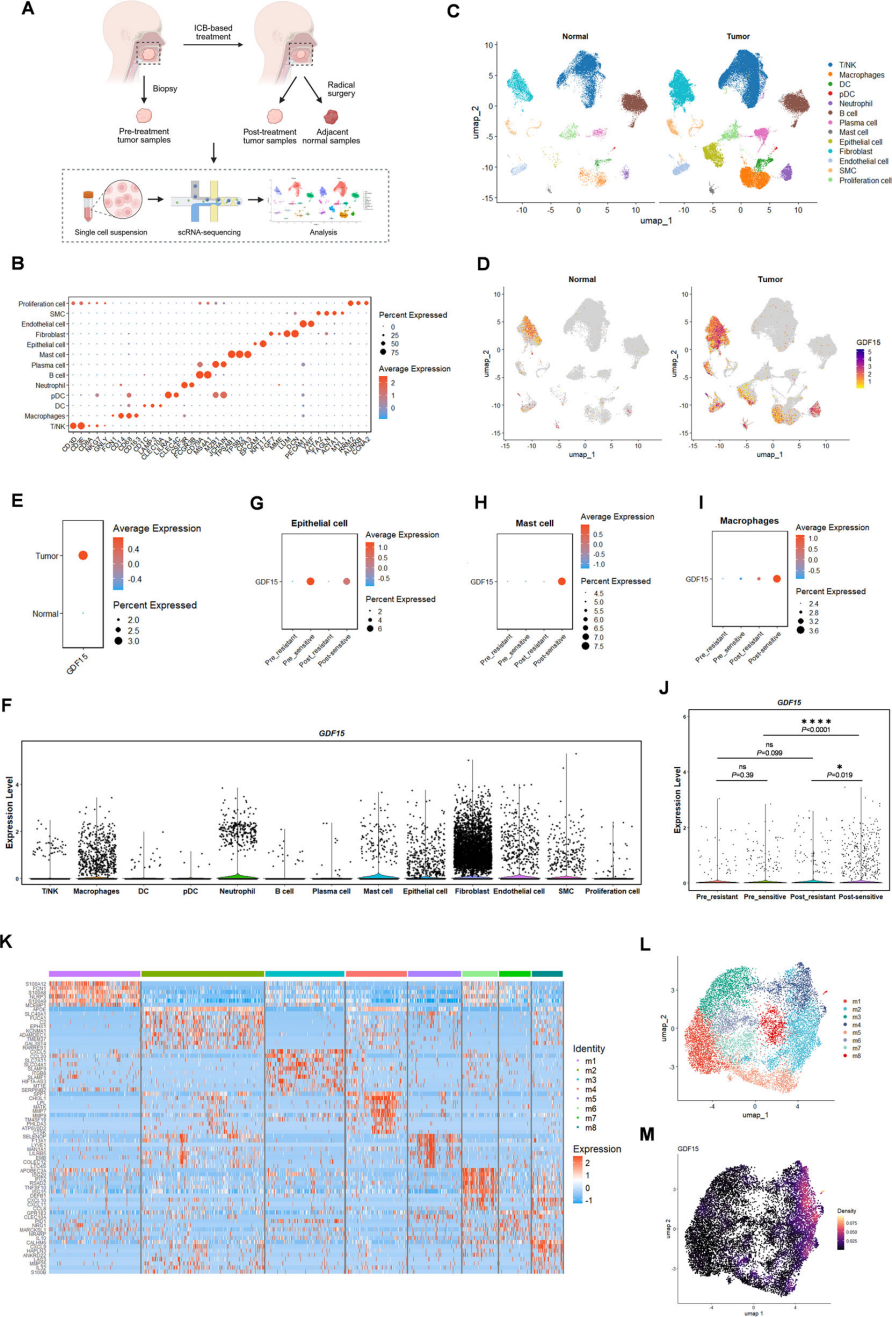
GDF15^+^ TAMs were enriched in OSCCs sensitive to ICB‐based therapy. (A) Schematic diagram showing the sample collection and scRNA‐seq process in this study. (B) Dot plot showing the markers for each cell clusters. (C) UMAP plots of 13 cell clusters in tumor and normal tissues. (D,E) Feature plots (D) and dot plot (E) comparing the expression of *GDF15* in normal and tumor tissues. (F) Violin plot of the expression level of *GDF15* in 13 cell clusters in tumor tissues. (G–I) Dot plots comparing the expression level of *GDF15* in the four groups (pre‐resistant, pre‐sensitive, post‐resistant, post‐sensitive) in epithelial cells (G), mast cells (H), and macrophages (I) in tumor tissues. (J) Violin plot comparing the expression level of *GDF15* in the four groups (pre‐resistant, pre‐sensitive, post‐resistant, post‐sensitive) in macrophages in tumor tissues. Statistical significance was analyzed via Kruskal–Wallis test. (K) Heatmap showing the markers for 8 clusters of macrophages in tumor tissues. (L) UMAP plot of 8 clusters of macrophages in tumor tissues. (M) Density plot showing the expression level of *GDF15* in 8 clusters of macrophages in tumor tissues. ^*^ *p* < 0.05, ^****^ *p* < 0.0001.

We previously collected 28 malignant samples, 5 marginal samples, and 9 normal samples from 19 OSCC patients who experienced ICB‐based therapy for scRNA‐seq, containing oral tissues and lymph nodes pre and post treatment (Figure 1A). The cells were divided into 13 clusters via the uniform manifold approximation and projection (UMAP) algorithm (Figure 1B, C). We first compared the expression levels of *GDF15* between malignant and normal samples and discovered that the expression of *GDF15* was significantly upregulated in tumor tissues, which was consistent with previous reports [14, 23] (Figure 1D, E).

To elucidate how GDF15 could affect the efficacy of ICB‐based therapy, we first assessed the expression of *GDF15* in different cell subtypes within tumor tissues. As illustrated in Figure 1F, macrophages (*FCN1* ^+^ *CD14* ^+^ *CD68* ^+^ *CD163* ^+^), mast cells (*TPSAB1* ^+^ *TPSB2* ^+^ *CPA3* ^+^), neutrophils (*CSF3R* ^+^ *FCGR3B* ^+^), endothelial cells (*PECAM1* ^+^ *VWF* ^+^), epithelial cells (*EPCAM* ^+^ *KRT17* ^+^) and fibroblasts (*FGF7* ^+^ *MME* ^+^ *LUM* ^+^ *DCN* ^+^) exhibited relatively higher levels of *GDF15* expression in the TME. We further evaluated the treatment response according to RECIST 1.1 criteria [24] and categorized the tumor samples into four groups (pre‐sensitive, pre‐resistant, post‐sensitive, post‐resistant) based on treatment response and timepoint of sampling. *GDF15* expression levels across these groups were then compared within each cell subtype. In fibroblasts (Figure S1A), neutrophils (Figure S1B), and endothelial cells (Figure S1C), *GDF15* was predominantly expressed in samples resistant to ICB‐based therapy. Thus, we speculated that the three cell subtypes were the main components that contributed to the synergistical antitumor effects of GDF15 neutralizing antibody on ICB‐based therapy. Conversely, in epithelial cells (Figure 1G), mast cells (Figure 1H), and macrophages (Figure 1I), *GDF15* expression was enriched in samples sensitive to ICB‐based therapy. While epithelial cells did not show substantial differences of *GDF15* expression before and after treatment (Figure 1G), mast cells (Figure 1H) and macrophages (Figure 1I) exhibited significantly elevated *GDF15* expression in post‐treatment samples that were sensitive to the therapy, indicating that the *GDF15*‐expressing mast cells and macrophages might help with tumor regression when treated with PD‐1 antibody. We speculated that the different expression patterns of *GDF15* might be representative of different functions of GDF15 in each cell subtype, which might underlie the “double‐edged sword” of GDF15 and limit the therapeutic effect of GDF15‐neutralizing antibody.

Since we expected to identify the potential factors that might hinder the therapeutic effect of the GDF15 neutralizing antibody, we focused on mast cells and macrophages in this study, both of which showed increased *GDF15* expression in post‐sensitive samples. Since the numbers of both total cells and specific *GDF15* ^+^ cells were greater in macrophages than in mast cells within the TME (Figure 1F), we prioritized *GDF15* ^+^ macrophages (cluster of macrophages, *GDF15* ^+^) for further exploration. The existence of GDF15^+^ TAMs subpopulation was verified by flow cytometry (Figure S1D–F). Figure 1J showed the expression level of *GDF15* specifically in TAMs. It turned out that *GDF15* expression level in TAMs did not differ significantly between resistant and sensitive pre‐treatment samples (*p* = 0.39), or between resistant samples before or after treatment (*p* = 0.099). However, the expression level of *GDF15* was significantly elevated in the sensitive samples after treatment (*p* < 0.0001), and was also higher compared with resistant samples after treatment (*p* = 0.019). Consistently, the proportion of *GDF15* ^+^ TAMs relative to total TAMs was increased by approximately two‐fold in responders compared with non‐responders after ICB‐based treatment. TAMs were further divided into eight clusters (m1–m8) (Figure 1K, L), with *GDF15* expression predominantly observed in m2 and m4 clusters (Figure 1M). Collectively, the results demonstrated a clinical association between increased enrichment of *GDF15* ^+^ TAMs and favorable response to ICB‐based treatment in OSCC.

### 2.2 GDF15 Deficiency in TAMs Accelerates Tumor Growth

Since human transcriptomic analysis revealed a clinical correlation between *GDF15* ^+^ TAMs and response to ICB, further exploration was required to elucidate the functional role of *GDF15* ^+^ TAMs in tumor progression. Due to the broad expression of GDF15 across multiple cell types, a genetic model that selectively alters its expression within the macrophages was necessary to determine the macrophage‐intrinsic role of GDF15 in TAMs. The *Lyz2*‐Cre system is a well‐established genetic tool for gene deletion in bone marrow‐derived myeloid cells and has been extensively validated for interrogating gene functions within bone marrow‐derived macrophages (BMDMs) in vivo [25]. Given that bone marrow‐derived monocytes constitute the major source of TAMs, we generated *Gdf15* ^fl/fl^ mice and *Gdf15* ^fl/fl^ *Lyz2* ^Cre^ mice (hereafter, *Gdf15* ^cKO^ mice) to investigate the role of GDF15 in macrophages during tumor development (Figure S2A). ELISA validated the knockout efficiency in BMDMs, showing that GDF15 was detected at approximately 500 pg/mL in the culture supernatant of *Gdf15* ^fl/fl^ BMDMs, whereas it was undetectable in the supernatant of *Gdf15* ^KO^ BMDMs (Figure S2B).

**FIGURE 2:**
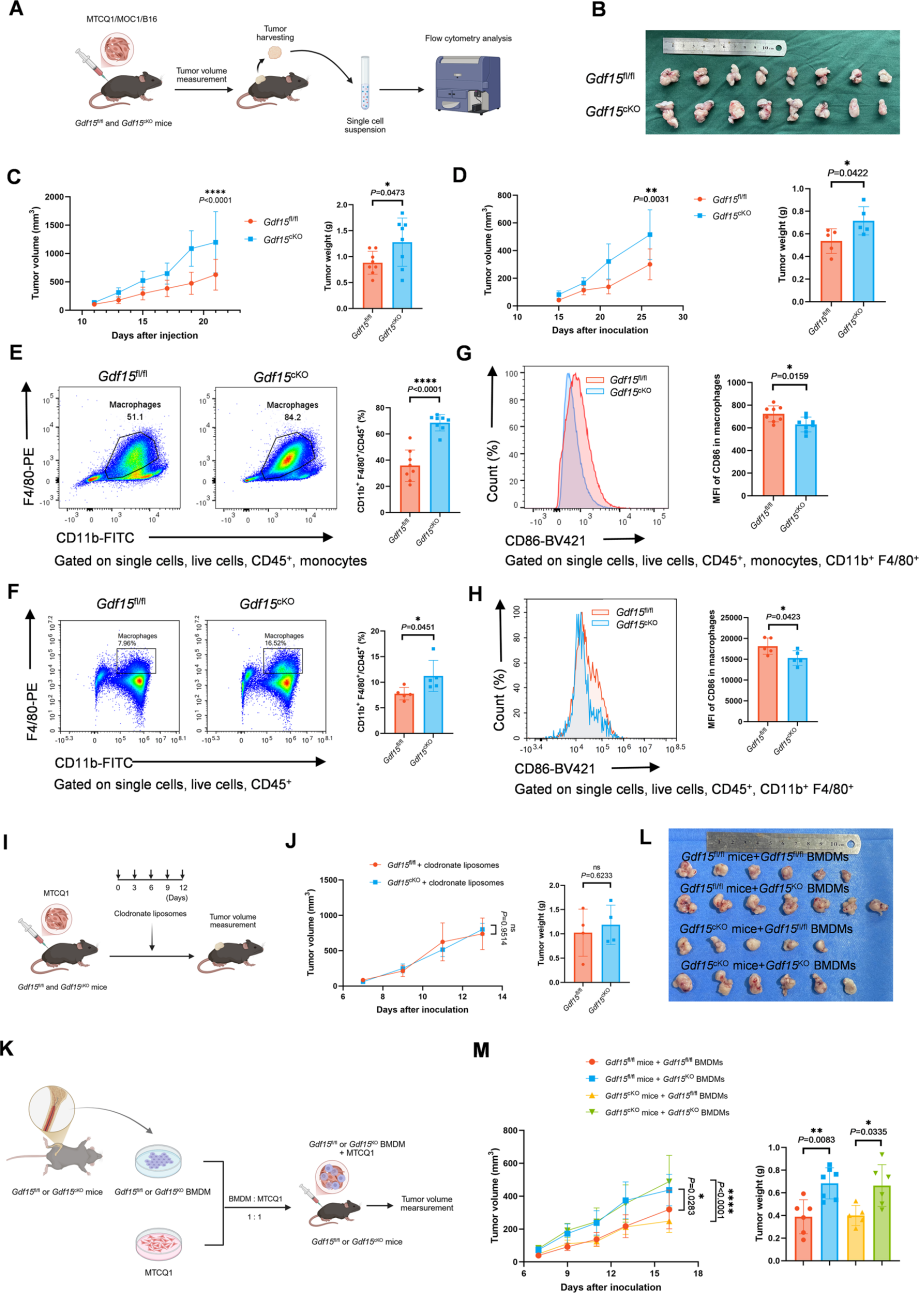
GDF15 deficiency in TAMs accelerates tumor growth. (A) Schematic diagram showing the process of subcutaneous tumor establishment and flow cytometry analysis. (B) Image of tumors dissected from MTCQ1‐tumor‐bearing *Gdf15* ^fl/fl^ and *Gdf15* ^cKO^ mice (*n* = 8). (C,D) Tumor volume curves and tumor weight of the MTCQ1 (*n* = 8) (C) and MOC1 (*n* = 5) (D) tumors in *Gdf15* ^fl/fl^ and *Gdf15* ^cKO^ mice. (E,F) Representative plots and statistical analysis showing the percentage of macrophages in the TME of MTCQ1 (E) and MOC1 (F) tumors in *Gdf15* ^fl/fl^ and *Gdf15* ^cKO^ mice detected by flow cytometry. (G–H) Representative plots and statistical analysis showing the expression of CD86 in the macrophages in the TME of MTCQ1 (G) and MOC1 (H) tumors in *Gdf15* ^fl/fl^ and *Gdf15* ^cKO^ mice detected by flow cytometry. (I) Schematic diagram showing the systemic clearance of macrophages during the process of subcutaneous tumor establishment. (J) Tumor volume curves and tumor weight of the MTCQ1 tumors in *Gdf15* ^fl/fl^ and *Gdf15* ^cKO^ mice when macrophages were systemically cleared in the meantime (*n* = 4). (K) Schematic diagram showing the process of establishing MTCQ1 subcutaneous tumors with the local supplement of *Gdf15* ^fl/fl^ or *Gdf15* ^cKO^ BMDMs. (L) Image of tumors dissected from MTCQ1‐tumor bearing *Gdf15* ^fl/fl^ and *Gdf15* ^cKO^ mice with local supplement of *Gdf15* ^fl/fl^ or *Gdf15* ^cKO^ BMDMs (*n* = 5–7). (M) Tumor volume curves and tumor weight of the tumors in the four groups. Statistical significance was analyzed via unpaired Student's *t*‐test (C right, D right, E‐H, J right), two‐way ANOVA followed by Tukey's multiple comparisons test (C left, D left, J left, M left), and one‐way ANOVA (M right). ^*^ *p* < 0.05, ^**^ *p* < 0.01, ^****^ *p* < 0.0001. Data were presented as mean ± SD. MFI, median fluorescence intensity.

Subcutaneous tumors of MTCQ1 and MOC1 cells were established in *Gdf15* ^fl/fl^ and *Gdf15* ^cKO^ mice (Figure 2A). Compared with *Gdf15* ^fl/fl^ mice, *Gdf15* ^cKO^ mice exhibited significantly increased tumor volumes and tumor weights, indicating that GDF15 deficiency in monocyte‐derived macrophages could accelerate tumor growth in mice (Figure 2B–D; Figure S2C). Subcutaneous tumors of B16 were also established to preliminarily explore whether the protumor effect of GDF15 deficiency was a universal phenomenon in other types of cancer besides OSCC. It was discovered that *Gdf15* ^cKO^ mice also endured accelerated tumor growth in melanoma compared with *Gdf15* ^fl/fl^ mice (Figure S2D,E).

We next characterized the TAMs in subcutaneous MTCQ1 and MOC1 tumors using flow cytometry. No significant difference was observed in the proportion of CD45^+^ immune cells between tumors in *Gdf1*5^fl/fl^ and *Gdf15* ^cKO^ mice (Figure S2F–H). A substantial increase in the proportion of CD11b^+^F4/80^+^ macrophages within CD45^+^ immune cells was observed in the tumors of *Gdf15* ^cKO^ mice (Figure 2E, F). However, TAMs from *Gdf15* ^cKO^ mice exhibited significantly lower expression levels of CD86, suggesting a weaker activation status of *Gdf15* ^KO^ TAMs (Figure 2G, H). Comparable expression levels of CD163 and PD‐L1 were observed in *Gdf15* ^fl/fl^ and *Gdf15* ^KO^ TAMs (Figure S2G,H).

To further confirm the protumor role of *Gdf15* ^KO^ TAMs, macrophages were systemically depleted in *Gdf15* ^fl/fl^ and *Gdf15* ^cKO^ mice via intraperitoneal (*i.p.)* injection of clodronate liposomes (Figure 2I). The efficacy of macrophage depletion was verified by flow cytometry (Figure S2I). Following depletion, the differences in tumor growth were abrogated between *Gdf15* ^fl/fl^ and *Gdf15* ^cKO^ mice (Figure 2J). Moreover, co‐inoculation of MTCQ1 and *Gdf15* ^KO^ BMDMs would significantly promote tumor growth in *Gdf15* ^fl/fl^ mice, whereas co‐inoculation of MTCQ1 and *Gdf15* ^fl/fl^ BMDMs would significantly suppress tumor growth in *Gdf15* ^cKO^ mice (Figure 2K–M). Overall, these results indicated that GDF15 deficiency in TAMs contributed to the acceleration of tumor growth.

### 2.3 GDF15 Deficiency in TAMs Suppresses Tumor Infiltration of CD8^+^ T Cells

**FIGURE 3:**
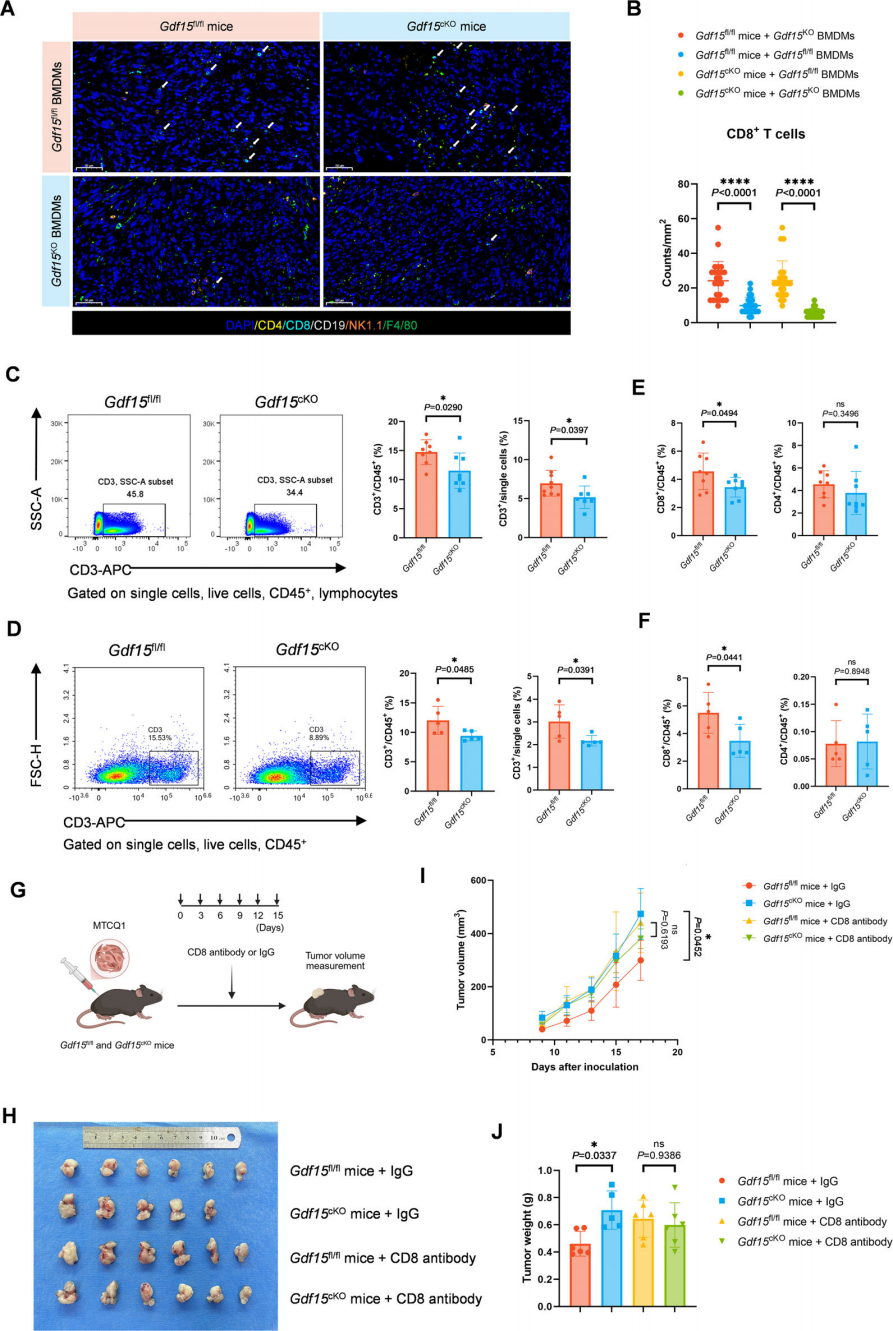
GDF15 deficiency in TAMs suppresses tumor infiltration of CD8^+^ T cells. (A) Representative mIHC staining images of the tumor samples in Figure 2L. The white arrows pointed out the tumor‐infiltrating CD8^+^ T cells. Scale bar, 50 µm. (B) Statistical analysis of the density of CD8^+^ T cells in the TME of the four groups detected by mIHC. (C,D) Representative plots and statistical analysis showing the percentage of CD3^+^ T cells in the TME of MTCQ1 (C) and MOC1 (D) tumors in *Gdf15* ^fl/fl^ and *Gdf15* ^cKO^ mice detected by flow cytometry. (E,F) Statistical analysis showing the percentages of CD4^+^ and CD8^+^ T cells in the TME of MTCQ1 (E) and MOC1 (F) tumors in *Gdf15* ^fl/fl^ and *Gdf15* ^cKO^ mice detected by flow cytometry. (G) Schematic diagram showing the systemic blockade of CD8^+^ T cells during the process of subcutaneous tumor establishment. (H) Image of tumors dissected from MTCQ1‐tumor‐bearing *Gdf15* ^fl/fl^ and *Gdf15* ^cKO^ mice injected *i.p*. with CD8 antibody or IgG in the meantime (*n* = 6). (I,J) Tumor volume curves (I) and tumor weight (J) of the MTCQ1 tumors in *Gdf15* ^fl/fl^ and *Gdf15* ^cKO^ mice when the mice were injected *i.p*. with CD8 antibody or IgG. Statistical significance was analyzed via unpaired Student's *t*‐test (C–F), two‐way ANOVA followed by Tukey's multiple comparisons test (I), and one‐way ANOVA (B, J). ^*^ *p* <0.05, ^****^ *p* <0.0001. Data were presented as mean ± SD.

To investigate whether GDF15^+^ TAMs could influence other immune cell populations in TME, we performed multiplex immunohistochemical (mIHC) staining on the MTCQ1‐BMDMs co‐inoculated tumors mentioned above (Figure 3A, B; Figure S3A–D). It illustrated that *Gdf15* ^KO^ BMDMs could significantly restrain CD8^+^ T cell infiltration and promote macrophage infiltration in *Gdf15* ^fl/fl^ mice, while *Gdf15* ^fl/fl^ BMDMs could significantly promote CD8^+^ T cell infiltration and restrain macrophage infiltration in *Gdf15* ^KO^ mice (Figure 3A, B; Figure S3A). GDF15 deficiency in TAMs failed to have any significant impact on the infiltration of CD4^+^ T cells, CD19^+^ B cells, or NK cells (Figure S3B–D).

Flow cytometry analysis of subcutaneous MTCQ1 and MOC1 tumors also demonstrated a significant reduction in the proportion of CD3^+^ T cells in *Gdf15* ^cKO^ mice compared with *Gdf15* ^fl/fl^ mice (Figure 3C, D). Among T cell subsets, GDF15 deficiency in TAMs significantly decreased the proportion of CD8^+^ T cells, but not CD4^+^ T cells, within CD45^+^ cells in the tumors of *Gdf15* ^cKO^ mice, suggesting that it was mainly the reduction of CD8^+^ T cell infiltration that contributed to the decrease of CD3^+^ T cells (Figure 3E, F). Expression levels of PD‐1, TIM3, and Granzyme B in CD8^+^ T cells were comparable between the two groups (Figure S3F). In addition, a lung metastasis model established with the B16 cell line also showed significantly impaired CD8^+^ T cell infiltration in *Gdf15* ^cKO^ mice (Figure S3G,H). The results above demonstrated that GDF15 deficiency in TAMs could suppress tumor infiltration of CD8^+^ T cells.

To further investigate whether GDF15^+^ TAMs indeed exert antitumor effects through inhibiting CD8^+^ T cells, *Gdf15* ^fl/fl^ and *Gdf15* ^cKO^ mice were treated with *i.p*. injection of CD8 antibody, which was verified to effectively eliminate CD8^+^ T cells in the spleen and the tumor in mice (Figure 3G; Figure S3I). It turned out that the difference in tumor growth between *Gdf15* ^fl/fl^ and *Gdf15* ^cKO^ mice were abolished by CD8 antibody (Figure 3H–J), suggesting that GDF15^+^ TAMs could mediate antitumor effects via promoting CD8^+^ T cell infiltration, while the mechanism of cellular interaction between CD8^+^ T cells and GDF15^+^ TAMs required further research.

### 2.4 Intrinsic GDF15 Enhances Phagocytosis and Antigen Cross‐Presentation of Macrophages

**FIGURE 4:**
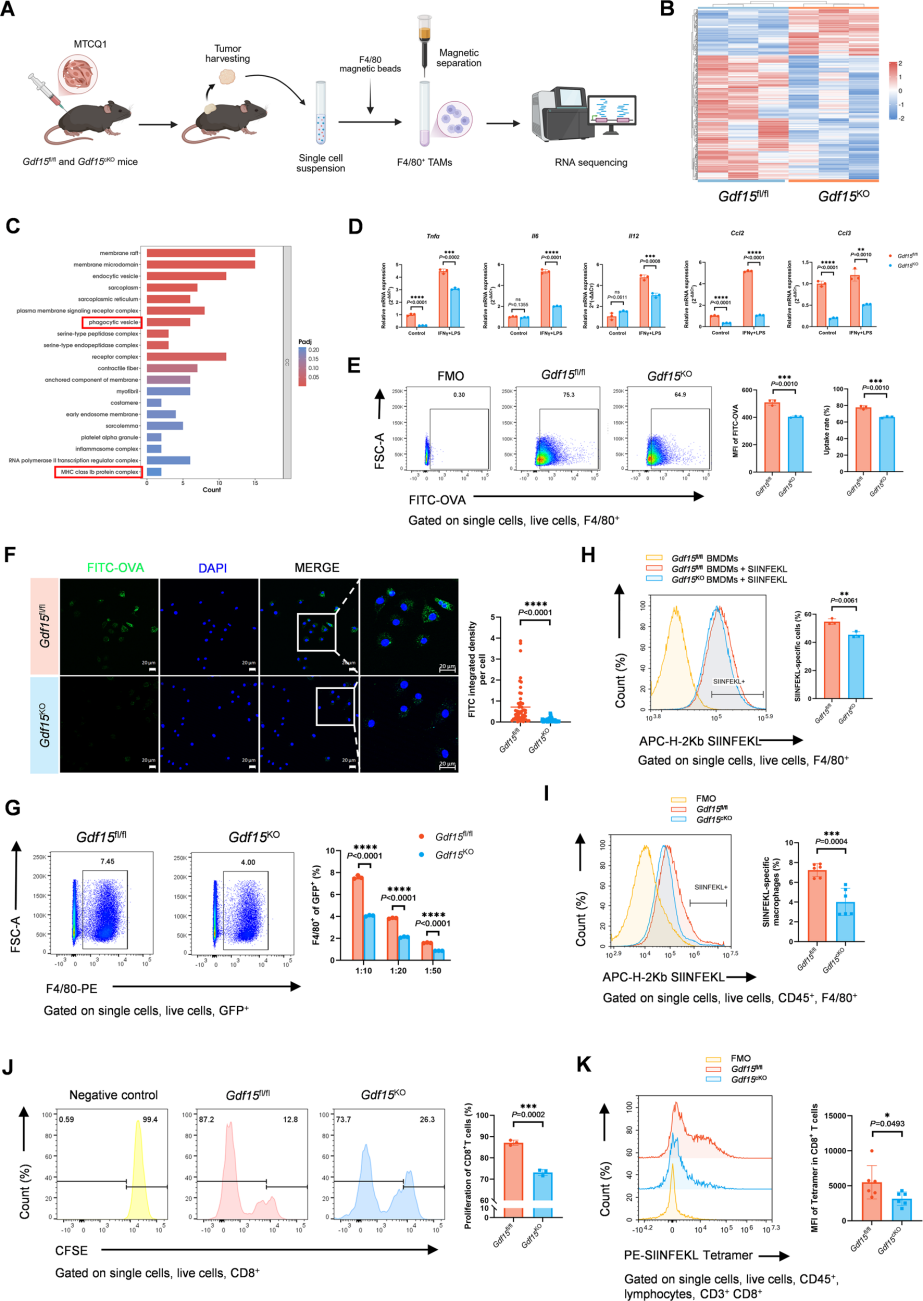
GDF15 enhances phagocytosis and antigen cross‐presentation of macrophages. (A) Schematic diagram showing the isolation and RNA‐seq analysis of *Gdf15* ^fl/fl^ and *Gdf15* ^KO^ TAMs. (B) Heatmap of the DEGs in *Gdf15* ^fl/fl^ and *Gdf15* ^KO^ TAMs (*n* = 3). (C) Bar plot showing the top 20 down‐regulated terms enriched by the GO analysis of CC in *Gdf15* ^KO^ TAMs. The red frames marked out the phagocytosis and antigen‐presentation related terms. (D) Real‐time PCR showing the mRNA expression of some M1‐polarization‐related pro‐inflammatory cytokines in *Gdf15* ^fl/fl^ and *Gdf15* ^KO^ BMDMs. (E) Representative plots and statistical analysis showing the percentage of macrophages participated in the phagocytosis of FITC‐OVA and MFI of the phagocytosed FITC‐OVA detected by flow cytometry. (F) Representative images and statistical analysis showing the phagocytosis of FITC‐OVA by *Gdf15* ^fl/fl^ and *Gdf15* ^KO^ BMDMs observed by confocal microscopy. Scale bar, 20 µm. (G) Representative plots and statistical analysis showing the percentage of MOC1‐GFP cells being phagocytosed by *Gdf15* ^fl/fl^ and *Gdf15* ^KO^ BMDMs detected by flow cytometry. (H) Representative plot and statistical analysis showing the percentage of *Gdf15* ^fl/fl^ and *Gdf15* ^KO^ BMDMs that could present H‐2Kb SIINFEKL after treated with 20 µg/mL of SIINFEKL peptide for 2 h detected by flow cytometry. (I) Representative plot and statistical analysis showing the percentage of TAMs that could present H‐2Kb SIINFEKL in the MTCQ1‐OVA‐tumor bearing *Gdf15* ^fl/fl^ and *Gdf15* ^cKO^ mice detected by flow cytometry (*n* = 6). (J) Representative plots and statistical analysis showing the proliferation of OT‐1 mice‐derived naïve CD8^+^ T cells stimulated by the *Gdf15* ^fl/fl^ and *Gdf15* ^KO^ BMDMs preloaded with SIINFEKL detected by flow cytometry. (K) Representative plots and statistical analysis showing the MFI of H2Kb/β2M/OVA (SIINFEKL) tetramer in CD8^+^T cells in subcutaneous MTCQ1‐OVA tumors in *Gdf15* ^fl/fl^ and *Gdf15* ^cKO^ mice (*n* = 6). Statistical significance was analyzed via an unpaired Student's *t*‐test. ^*^ *p* < 0.05, ^**^ *p* < 0.01, ^***^ *p* < 0.001, ^****^ *p* < 0.0001. Data were presented as mean ± SD.

To further explore the mechanism underlying the tumor suppressive effect of GDF15^+^ TAMs, F4/80^+^ macrophages were isolated from the subcutaneous MTCQ1 tumors established in *Gdf15* ^fl/fl^ and *Gdf15* ^cKO^ mice using magnetic beads. RNA‐sequencing (RNA‐seq) analysis was subsequently performed on the isolated *Gdf15* ^fl/fl^ and *Gdf15* ^KO^ TAMs (Figure 4A, B). The purity of the isolated F4/80^+^ cells was confirmed by flow cytometry before sequencing (Figure S4A). RNA‐seq identified 143 upregulated and 384 downregulated genes, including *Lyz2* and *Gdf15*, which incidentally verified the knock‐out efficacy of *Gdf15* in TAMs (Figure S4B). Gene Ontology (GO) enrichment concerning cellular component (CC) and Kyoto Encyclopedia of Genes and Genomes (KEGG) enrichment of differentially expressed genes (DEGs) revealed significant reduction in “phagocytic vesicle”, “MHC class Ib protein complex” (Figure 4C) and “phagosome” (Figure S4C) in *Gdf15* ^KO^ TAMs, suggesting that *Gdf15* ^KO^ TAMs might have diminished phagocytic and antigen cross‐presentation abilities compared with *Gdf15* ^fl/fl^ TAMs. To verify whether GDF15 could have the same effect on TAMs in human OSCC, KEGG enrichment analysis of the upregulated DEGs in *GDF15* ^+^ TAMs vs. *GDF15* ^−^ TAMs in scRNA‐seq was also performed and showed a significant upregulation of the “phagosome” pathway in *GDF15* ^+^ TAMs, which was in accordance with the results of murine TAMs (Figure S4D). Moreover, GO enrichment analysis of biological process (BP) and molecular function (MF) indicated impaired cytokine‐related activities in *Gdf15* ^KO^ TAMs (Figure S4E,F), which was also supported by the decreased expression of several M1‐polarization‐related pro‐inflammatory cytokines validated by real‐time PCR (Figure 4D). These findings indicated that *Gdf15* ^KO^ TAMs might exhibit a general reduction in immune functions.

We next performed a series of in vitro and in vivo assays to detect whether GDF15 could enhance phagocytosis in macrophages. First, FITC‐ovalbumin (OVA) was used as a classic probe to measure phagocytic ability. Flow cytometry analysis illustrated that *Gdf15* ^fl/fl^ BMDMs could uptake a significantly higher rate of FITC‐OVA compared with *Gdf15* ^KO^ BMDMs (77.73% vs 66.03%, *p* = 0.0010) (Figure 4E). Both flow cytometry analysis (Figure 4E) and confocal microscopy (Figure 4F) showed a significantly higher fluorescence intensity of FITC‐OVA in *Gdf15* ^fl/fl^ BMDMs compared with *Gdf15* ^KO^ BMDMs. Furthermore, *Gdf15* ^fl/fl^ BMDMs could also phagocytose more MOC1‐GFP cells than *Gdf15* ^KO^ BMDMs at various co‐culture ratios (Figure 4G). For in vivo validation, MOC1‐GFP was subcutaneously injected, and F4/80^+^GFP^+^ cells were identified as TAMs that had recently phagocytosed tumor cells. Consistent with the in vitro results, *Gdf15* ^fl/fl^ TAMs also showed higher phagocytic rates of MOC1‐GFP cells compared with *Gdf15* ^KO^ TAMs (Figure S4G). The results above confirmed that GDF15 could enhance the phagocytic ability of macrophages.

Interestingly, we observed a significant higher apoptosis rate of MOC1 cells when they were co‐cultured with *Gdf15* ^fl/fl^ BMDMs rather than *Gdf15* ^KO^ BMDMs (Figure S4H). Confocal microscopy further revealed that, when CFSE‐labeled MOC1 cells were co‐cultured with either *Gdf15* ^fl/fl^ or *Gdf15* ^KO^ BMDMs at the same ratio, more intact MOC1 cells with preserved morphology remained in the system when co‐cultured with *Gdf15* ^KO^ BMDMs, indicating that GDF15 deficiency would weaken the tumor‐eliminating capability of macrophages (Figure S4I).

Given the reduced MHC I‐related antigen presentation of *Gdf15* ^KO^ TAMs and the decreased proportion of CD8^+^ T cells observed in tumors from *Gdf15* ^cKO^ mice, we speculated that GDF15 might influence CD8^+^ T cell infiltration by modulating the antigen cross‐presentation capacity of macrophages. We first assessed the ability of macrophages to present antigens through MHC I molecules. An in vitro antigen presentation assay showed that *Gdf15* ^fl/fl^ BMDMs could present more H2‐Kb SIINFEKL complexes than *Gdf15* ^KO^ BMDMs after 2 h of SIINFEKL peptide treatment, exhibiting 54.74% of H2Kb‐SIINFEKL^+^ cells in *Gdf15* ^fl/fl^ BMDMs while 45.43% of *Gdf15* ^KO^ BMDMs (Figure 4H). Consistently, TAMs from MTCQ1‐OVA tumor‐bearing *Gdf15* ^fl/fl^ mice also displayed a greater capacity for H2‐Kb SIINFEKL complex presentation in vivo compared with TAMs from *Gdf15* ^cKO^ mice (Figure 4I; Figure S4J,K). We then evaluated the activation of tumor‐infiltrating CD8^+^T cells in response to antigen‐specific macrophages. *Gdf15* ^fl/fl^ and *Gdf15* ^KO^ BMDMs were pretreated with SIINFEKL peptides and co‐cultured with CFSE‐labeled naïve CD8^+^ T cells from OT‐1 mice. Flow cytometry analysis detected an enhanced ability of *Gdf15* ^fl/fl^ BMDMs to stimulate CD8^+^ T cell proliferation compared with *Gdf15* ^KO^ BMDMs (Figure 4J). The MFI of H2Kb/β2M/OVA (SIINFEKL) tetramer in CD8^+^T cells and the frequency of tumor‐infiltrating SIINFEKL‐specific CD8^+^T cells in MTCQ1‐OVA subcutaneous tumors were also significantly higher in *Gdf15* ^fl/fl^ mice (7.23%) than in *Gdf15* ^cKO^ mice (4.01%) (Figure 4K), while the expression of CD25 and CD69 in CD8^+^T cells showed no significant differences between the two groups (Figure S4L). Collectively, GDF15 in macrophages could enhance the ability of antigen cross‐presentation to CD8^+^ T cells.

### 2.5 GDF15 Could Locate in the Nucleus of Macrophages But Does Not Regulate the Functions in the Form of a Transcription Factor

Next, we attempted to investigate how GDF15 could promote the phagocytic and antigen‐presenting abilities of macrophages. GDNF family receptor α‐like protein (GFRAL) is so far the only well‐recognized membrane receptor known to bind to secreted GDF15 [26, 27, 28]. However, GFRAL is exclusively expressed in neurons of the area postrema and nucleus of the solitary tract in mice and human [29]. Consistently, scRNA‐seq analysis of OSCC samples also confirmed that none of the cells in the TME could express GFRAL (Figure S5A,B), suggesting that GDF15 expressed in the TME is unlikely to influence tumor progression through binding to GFRAL.

Previous studies have reported that GDF15 could enter the cell nucleus of tumor cells and regulate multiple signaling pathways, generally exerting antitumor effects [30, 31, 32]. In this study, nuclear localization of GDF15 was also observed in macrophages using confocal microscopy (Figure S5C) and western blotting (Figure S5D). The overall and nuclear expression levels of GDF15 were not affected by the polarization state of macrophages. The major band of GDF15 at 35 kDa was detected in the nuclear protein under reducing condition, which likely corresponded to the GDF15 precursor monomer. This was distinct from the mature GDF15 monomer at 15 kDa observed in total cell lysates under reducing condition (Figure S5D), indicating that GDF15 may exert its biological functions in its precursor form in the nucleus rather than the mature form like secreted GDF15.

Furthermore, we performed Chromatin Immunoprecipitation‐sequencing (ChIP‐seq) in the THP‐1‐derived macrophages to explore whether GDF15 could act as a transcription factor in the nucleus. The results showed that GDF15 could bind to the promoter regions of 108 genes. However, none overlapped with the DEGs identified by RNA‐seq (Figure S5E,F). Taken together, these findings suggested that although GDF15 could localize in the nucleus of macrophages, it might not regulate the phagocytic or antigen‐presenting abilities in the form of a transcription factor.

### 2.6 Intrinsic GDF15 Regulates Macrophage Functions Through NF‐κB Pathway

**FIGURE 5:**
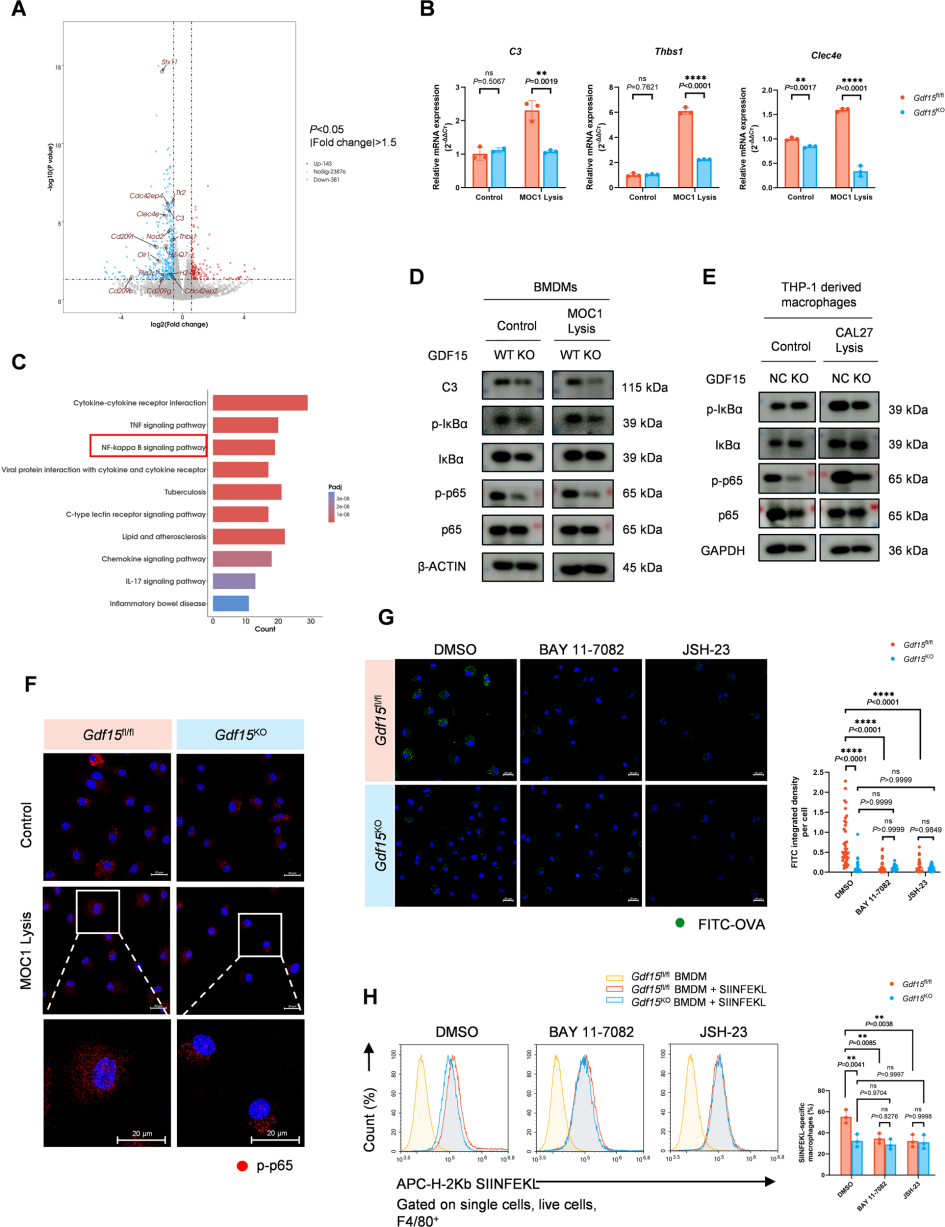
GDF15 regulates macrophage functions through the NF‐κB pathway. (A) Volcano plot of the DEGs between *Gdf15* ^fl/fl^ and *Gdf15* ^KO^ TAMs. *Gdf15*, *Lyz1*, and *Lyz2* were excluded in this plot. The down‐regulated genes enriched in the terms related to phagocytosis or antigen‐presentation in GO and KEGG analysis were marked out. (B) Real‐time PCR verification of the phagocytosis or antigen‐presentation related down‐regulated genes in *Gdf15* ^KO^ TAMs screened out by RNA‐seq. The genes whose mRNA expression was confirmed to be significantly downregulated in *Gdf15* ^KO^ BMDMs were presented. (C) Bar plot showing the top 10 down‐regulated pathways enriched by the KEGG analysis in *Gdf15* ^KO^ TAMs. The NF‐κB signaling pathway ranked third and was marked out with a red frame. (D) Western blotting analysis of NF‐κB signaling pathway in *Gdf15* ^fl/fl^ and *Gdf15* ^KO^ BMDMs treated with or without MOC1 cell lysis for 24 h. (E) Western blotting analysis of NF‐κB signaling pathway in THP‐1‐derived macrophages treated with or without CAL27 cell lysis for 24 h. (F) Representative images of immunofluorescence staining of p‐p65 in *Gdf15* ^fl/fl^ and *Gdf15* ^KO^ BMDMs observed by confocal microscopy. Scale bar, 20 µm. (G) Representative confocal images and statistical analysis showing the phagocytosis of FITC‐OVA by *Gdf15* ^fl/fl^ and *Gdf15* ^KO^ BMDMs when treated with BAY 11‐7082 (1 µM) or JSH‐23 (20 µM). Scale bar, 20 µm. (H) Representative plot and statistical analysis showing the percentage of *Gdf15* ^fl/fl^ and *Gdf15* ^KO^ BMDMs that could present H‐2Kb SIINFEKL after treated with 20 µg/mL of SIINFEKL peptide for 2 h with the existence of BAY 11‐7082 (1 µM) or JSH‐23 (20 µM). Statistical significance was analyzed via unpaired Student's *t*‐test (B) and one‐way ANOVA (G, H). ^**^ *p* < 0.01, ^****^ *p* < 0.0001. Data were presented as mean ± SD.

Next, the DEGs identified through RNA‐seq and enriched in the phagocytosis and antigen presentation‐related GO and KEGG terms were selected for further investigation (Figure 5A). Real‐time PCR validation in BMDMs and THP‐1‐derived macrophages showed that *Complement 3* (*C3*) was the most significantly downregulated DEG in both murine *Gdf15* ^KO^ and human *GDF15* ^KO^ macrophages (Figure 5B; Figure S6A–C). KEGG enrichment analysis showed a significant downregulation of the NF‐κB signaling pathway in *Gdf15* ^KO^ TAMs (Figure 5C). Western blotting confirmed that the phosphorylation of p65 was significantly decreased in both *Gdf15* ^KO^ BMDMs (Figure 5D) and *GDF15* ^KO^ THP‐1‐derived macrophages (Figure 5E), under both unstimulated conditions and 24 h stimulation of OSCC cell lysis, whereas the total expression level of p65 remained unaffected. Less distribution of p‐p65 was also observed in *Gdf15* ^KO^ BMDMs by confocal microscopy (Figure 5F).

To confirm that it was the NF‐κB signaling pathway that mediated the promoting effects of GDF15 on phagocytic and antigen cross‐presenting functions of macrophages, we employed two NF‐κB pathway inhibitors for further investigation. BAY‐11‐7082 could irreversibly inhibit phosphorylation of IκBα [33] and JSH‐23 could inhibit transcription and nuclear translocation of NF‐κB [34]. Both BAY 11‐7082 and JSH‐23 could significantly weaken the ability of *Gdf15* ^fl/fl^ BMDMs to phagocytose FITC‐OVA (Figure 5G) and to present H2‐Kb SIINFEKL complex (Figure 5H), making it comparable with those of *Gdf15* ^KO^ BMDMs. However, neither inhibitor could further reduce the already impaired functions of *Gdf15* ^KO^ BMDMs. The similar effects of *Gdf15* gene knockout and NF‐κB blockade in GDF15‐expressing macrophages indicated that NF‐κB could act downstream of GDF15 when regulating the functions. Together, these results demonstrated that GDF15 could enhance phagocytosis and antigen cross‐presentation of macrophages through a NF‐κB signaling pathway‐dependent way.

## 3 Discussion

In this study, we performed scRNA‐seq on OSCC samples before and after ICB‐based treatment and identified GDF15^+^ TAMs as an important cell subset mediating tumor regression after ICB‐based treatment. *Gdf15* ^fl/fl^ *Lyz2* ^Cre^ mice were further utilized and demonstrated that GDF15 deficiency in macrophages could promote tumor growth by suppressing CD8^+^ T cell infiltration. Expression of GDF15 in macrophages could enhance phagocytosis and antigen cross‐presentation to CD8^+^ T cells through the NF‐κB signaling pathway, thereby inhibiting tumor progression.

Although GDF15 is regarded as a double‐edged sword in tumor regulation, most existing studies have focused on its oncogenic effects. In tumor cells, GDF15 has been reported to promote proliferation, migration, and invasion in various cancers [14, 15, 35]. In macrophages, where GDF15 was originally discovered and named MIC‐1, recombinant MIC‐1 was shown to inhibit the expression of TNF‐α stimulated by LPS [9]. Recombinant GDF15 was also reported to suppress the secretion of TNF‐α and NO by inhibiting the NF‐κB pathway, thereby facilitating the progression of pancreatic cancers [16]. In dendritic cells (DCs), recombinant GDF15 was found to inhibit DC maturation and migration by binding to CD44 on the cell surface, thus promoting immune escape in ovarian cancer [36]. Moreover, GDF15 could weaken the activation and antigen cross‐presentation of DC, consequently reducing the cytotoxic activity of CD8^+^ T cells [37]. In regulatory T (Treg) cells, GDF15 could bind to CD48 on the cell surface to promote Treg generation and enhance the suppressive functions, thereby establishing an immunosuppressive TME in hepatocellular carcinoma [38].

However, there were still some studies that obtained results highly consistent with our study. For example, microglia, as the innate immune cells in the central nervous system, are mainly generated from bone marrow‐derived monocytes and considered to be homologous to BMDMs. Recently, Chen et al. [39] reported that knockdown of *Gdf15* in the mouse microglial cell line BV2 could significantly inhibit its phagocytic ability, down‐regulate the NF‐κB signaling pathway, and reduce the expression of *Il‐6*, *Tnf‐α*, and *Ccl2* in response to LPS. The results obtained by *Gdf15* knockdown using siRNA were highly consistent with our results generated by CRISPR‐Cas9–mediated *Gdf15* knockout, while they were completely opposite to the studies that employed recombinant GDF15. Moreover, extracellular and intracellular GDF15 were also discovered to exert opposite regulatory effects on tumor cells. Intracellular GDF15 could inhibit PD‐L1 expression in gallbladder cancer, while extracellular GDF15 could promote its expression [40]. Therefore, we hypothesized that it was the mode of GDF15 intervention that determined the effect direction of GDF15 in macrophages. To verify this hypothesis, we treated *Gdf15* ^fl/fl^ and *Gdf15* ^KO^ BMDMs with recombinant GDF15 and found that both the knockout of *Gdf15* and recombinant GDF15 treatment could impair the phagocytic ability of macrophages. Moreover, the inhibitory effect of recombinant GDF15 protein on phagocytosis was more significant in *Gdf15* ^fl/fl^ BMDMs than in *Gdf15* ^KO^ BMDMs (Figure S7). These results confirmed our hypothesis that the mode of GDF15 intervention could critically affect its functional impact on macrophages, with intrinsic GDF15 exerting an antitumor role while the secreted form of GDF15 exerting a protumor role, and the latter could be neutralized by GDF15 antibody. Thus, we would like to propose a model where the net effect of GDF15 in the TME is a balance between its immunosuppressive, secreted form and its immunostimulatory, macrophage‐intrinsic (and possibly other cell subtypes‐intrinsic) form. The therapeutic efficacy of GDF15 neutralization may depend on ablating the former while sparing the latter. In this context, the antitumor effect of macrophage‐intrinsic GDF15 would not be neutralized by GDF15 antibody, but would instead be unmasked. Thus, our findings, although seemingly contrary to the clinical trial outcomes, may actually provide mechanistic evidence supporting the therapeutic effect of the GDF15 antibody that neutralize the secreted form of GDF15.

In 2006, Min et al. [31] first reported that GDF15 could enter the nucleus of osteosarcoma cells in its precursor form. Nuclear GDF15 was found to block the DNA‐binding activity of the SMAD complex, thereby inhibiting TGF‐β‐mediated cell invasion and EMT and exerting a tumor suppressive effect. Thereafter, Zhou et al. [30] reported that nuclear GDF15 could function as a transcriptional coactivator by interacting with specificity protein‐1 (Sp1) to upregulate KISS‐1 expression, thereby inhibiting the proliferation of breast cancer cells. These results shed light on a new approach to explain the double‐edged effect of GDF15, suggesting that the nuclear GDF15 precursor and the secreted mature GDF15 may carry out different biological functions in tumor regulation. Specifically, nuclear GDF15 tended to act as a tumor suppressor, whereas the mature secreted form more often functioned as a tumor promoter.

In this study, we also sought to explain the two opposite effects of GDF15 in macrophages from the perspective of distinct functions of extracellular vs. intracellular GDF15 as previously reported [40]. We performed ChIP‐seq in THP‐1‐ derived macrophages to assess whether GDF15 could influence phagocytosis and antigen presentation of macrophages by translocating into the nucleus and directly regulating gene expression. Unfortunately, we failed to identify any phagocytosis‐, antigen presentation‐, or NF‐κB pathway‐related genes whose expression could be directly regulated by GDF15 through binding to the promoter. The lack of specific molecular mechanisms represents one limitation of our study and further research is still warranted to explain this phenomenon.

Besides the enhancement of phagocytosis and antigen presentation, GDF15 could also promote the secretion of multiple pro‐inflammatory cytokines in macrophages, as revealed by the RNA‐seq results in this study. This might represent one of the mechanisms through which GDF15^+^ TAMs exert antitumor effects. The increased secretion of pro‐inflammatory cytokines could result from the coordinated regulation of GDF15 on the NF‐κB signaling pathway or from feedback interactions between phagocytosis and cytokine secretion. A limitation of this study is that we did not investigate the precise molecular mechanisms by which GDF15 could regulate cytokine expression to suppress tumor progression, and this phenomenon warrants further investigation in future studies.

In addition, although we discovered GDF15^+^ TAMs as a crucial subpopulation in mediating tumor regression in human OSCC samples, the subsequent functional and mechanistic exploration were mainly conducted in murine macrophages. The lack of validation in the relevance for human therefore, constitutes another limitation of this study.

In summary, the GDF15 neutralizing antibody has shown promising efficacy in several clinical trials for the treatment of cancer cachexia [41, 42] and solid tumors resistant to ICB therapy [8]. In this study, we discovered that GDF15^+^ TAMs were significantly enriched in the OSCCs responsive to ICB‐based treatment, which might mediate tumor regression after treatment. Further investigation revealed that macrophage‐intrinsic GDF15 could enhance phagocytosis and antigen cross‐presentation through the NF‐κB signaling pathway, thereby inhibiting tumor progression. Moreover, we discovered the opposite effect of secreted and intrinsic GDF15 in macrophages and proposed a model where the net effect of GDF15 in the TME is a balance between its immunosuppressive, secreted form and its immunostimulatory, macrophage‐intrinsic form. GDF15 neutralization could exert its therapeutic effect on ablating the former while sparing the latter. This study would help gain a more comprehensive understanding of the double‐edged effect of GDF15, thus ensuring a safer and more effective application of GDF15 neutralizing antibody in the future.

## 4 Methods

### 4.1 Mice

*Gdf15* ^fl/fl^ and *Gdf15* ^cKO^ mice were generated by GemPharmatech Co.,Ltd (Jiangsu, China) using CRISPR‐Cas9 technology. Exon 2 of the *Gdf15*‐201 transcript, which contains most of the coding sequence, was chosen as the knockout region. First, two sgRNAs targeting the introns on both sides of the floxed region of *Gdf15* were constructed and transcribed in vitro, and a donor vector containing the loxp fragment was designed and constructed. Then, Cas9, sgRNAs, and the donor were microinjected into zygotes of C57BL/6JGpt mice. The zygotes were transplanted to obtain positive F0 mice, which were confirmed by PCR and sequencing. The heterozygous F1 generation mice were obtained by mating F0 generation mice with C57BL/6JGpt mice. Further mating was implemented to obtain the homozygous *Gdf15* ^fl/fl^ mice. The *Gdf15* ^fl/fl^ mice were then mated with mice expressing *Lyz2*‐Cre. The *Gdf15* ^fl/fl^ *Lyz2* ^Cre^ mice (*Gdf15* ^cKO^ mice) were ultimately obtained to ablate the expression of *Gdf15* in myeloid cells. The OT‐1 mice were purchased from GemPharmatech Co.,Ltd (Jiangsu, China). All the mice were housed in the SPF facility of the Ninth People's Hospital, Shanghai Jiao Tong University, China. All the laboratory procedures were approved by the Laboratory Animal Care and Use Committee of the hospital (SH9H‐2024‐A418‐SB).

### 4.2 Cell Lines

The mouse OSCC cell line MTCQ1 and the mouse melanoma cell line B16 were cultured in DMEM with 10% fetal bovine serum (FBS) and 1% penicillin/streptomycin. The mouse OSCC cell line MOC1 was cultured in a medium mixed with IMDM (sh30228.02, HYclone, USA) and Ham's Nutrient Mixture F10‐F12 (sh30026.01, HYclone, USA) in a 2:1 ratio, with 10% FBS, 5 mg/L of insulin, 40 µg/L of hydrocortisone, 5 µg/L of epidermal growth factor (EGF) and 1% penicillin/streptomycin. The human monocytic leukemia cell line THP‐1 and the mouse macrophage cell line RAW 264.7 were cultured in RPMI 1640 with 10% FBS and 1% penicillin/streptomycin. All cells were cultured in a humidified atmosphere of 5% CO_2_ at 37°C.

### 4.3 Human Samples

28 malignant samples, 5 marginal samples, and 9 normal samples from 19 OSCC patients who underwent PD‐1 antibody‐related therapies were collected from Ninth People's Hospital, Shanghai Jiao Tong University, China with informed consent. All procedures were approved by the ethics committee of the hospital (SH9H‐2020‐T382‐9). The treatment responses were evaluated according to the Response Evaluation Criteria in Solid Tumors (RECIST), version 1.1 [24]. Patients with complete response (CR) or partial response (PR) were considered to be treatment‐sensitive while patients with progressive disease (PD) or stable disease (SD) were considered to be treatment‐resistant.

### 4.4 Identification of Mouse Genotypes

About 2 mm of the mouse tail tissue was cut for the identification of genotypes. The extraction of DNA and PCR were performed according to the protocol of the mouse tissue direct PCR kit (10189ES70, Yeasen, China). The primers were as follows: *Gdf15*‐flox forward 5’‐ AAGAACCTGCGGGAACTGGATC‐3’ and reverse 5’‐ CCGGAGTAGTTAGGATGGCAGAC‐3’; Lyz2‐iCre‐1 forward 5’‐ AGTGCTGAAGTCCATAGATCGG‐3’ and reverse 5’‐ CTGATTCTCCTCATCACCAGG‐3’; Lyz2‐iCre‐2 forward 5’‐ AGTGCTGAAGTCCATAGATCGG‐3’ and reverse 5’‐ GTCACTCACTGCTCCCCTGT‐3’. The primers were synthesized by Sangon Biotech (Shanghai, China). The PCR products were analyzed by agarose gel electrophoresis.

### 4.5 Isolation and Culture of BMDMs

The 6 to 8‐week‐old mice were sacrificed and dissected for femurs and tibias. Both ends of the bones were cut off using a sterilized scissor and the bone marrow cavities were flushed 3 times with pre‐cooled PBS in a 1 mL syringe. The flushed fluid was collected and centrifuged at 500 g for 5 min to obtain bone marrow cells. The cell pellet was resuspended and cultured in DMEM with 30 ng/mL of macrophage colony stimulating factor (M‐CSF, RPM0002, ABclonal, China), 10% FBS, and 1% penicillin/streptomycin (day 0). The culture medium was replaced on day 3. BMDMs were ready for subsequent experiments on day 5. M‐CSF (30 ng/mL) is required throughout the cultivation process of BMDMs. For harvesting BMDMs, the cells were rinsed and then treated with the EDTA digestion buffer (20% FBS and 10mM in PBS) for 5 min. The reaction was terminated by adding 4 volumes of PBS and the cell suspension was centrifuged at 500g for 5 min to obtain the BMDM pellet.

### 4.6 Tumor Models and Measurement

MTCQ1 (1 × 10^6^), MOC1 (1 × 10^6^), or B16 (5 × 10^5^) cells were inoculated subcutaneously into the right back of 6 to 8‐week‐old *Gdf15* ^fl/fl^ and *Gdf15* ^cKO^ mice. The tumor volumes were monitored throughout the process and were calculated with the formula of “volume = length×width^2^/2.” The mice were sacrificed for subsequent investigation before the tumor volumes reached 1500 mm^3^. For the lung metastasis model, B16 (1 × 10^6^) cells were injected intravenously (*i.v*.). Fourteen days after injection, the mice were sacrificed for subsequent experiments.

### 4.7 Systemic Clearance of Macrophages In Vivo

For systemic macrophage depletion, the mice were inoculated with subcutaneous tumors and were injected i.p. with 150 µL clodronate liposomes (40337ES08, Yeasen, China) on the same day (day 0). Afterward, clodronate liposomes were injected *i.p*. every 3 days (q3d) until the mice were sacrificed. The macrophage clearance efficacy in spleens and tumors was verified through flow cytometry analysis.

### 4.8 Local Supplementation of Macrophages in Tumors

MTCQ1 (1 × 10^6^) cells were mixed with an equal number of BMDMs from *Gdf15* ^fl/fl^ or *Gdf15* ^cKO^ mice and inoculated subcutaneously into the right back of 6 to 8‐week‐old *Gdf15* ^fl/fl^ and *Gdf15* ^cKO^ mice. The tumor volumes were measured throughout the process.

### 4.9 Systemic Blockade of CD8^+^ T Cells In Vivo

For systemic CD8^+^ T cells blockade, the mice were inoculated with a subcutaneous tumor and were injected *i.p*. with 100 µg anti‐mouse CD8α (A2102, clone 2.43, Selleck, USA) or the same amount of IgG2b isotype control (A2116, clone LTF‐2, Selleck, USA) on the same day (day 0). Afterward, the antibodies were injected *i.p*. q3d until the mice were sacrificed. The blockade efficacy in spleens and tumors was verified through flow cytometry analysis.

### 4.10 Preparation of Single‐Cell Suspension of Tumors

The tumors were first cut into small pieces with a diameter of 1–2 mm. The tumor fragments were then treated with 2 mg/mL of collagenase (C0130, Sigma–Aldrich, USA), 0.2 mg/mL of DNase (abs47047435, Absin, China), and 0.2 mg/mL of hyaluronidase (abs47014926, Absin, China) in RPMI 1640 medium and incubated at 37°C for 20 min. The digestion was terminated by adding twice the volume of RPMI 1640 medium with 10% FBS. The remaining tumor fragments were ground and the fluid was filtered through a 70 µm cell strainer to obtain a single‐cell suspension. The suspension would be used for subsequent flow cytometry analysis or magnetic isolation of TAMs.

### 4.11 Magnetic Isolation of TAMs

Anti‐F4/80 microbeads ultrapure (130‐110‐443, Miltenyi Biotec, USA) was used for magnetic isolation of TAMs in subcutaneous tumors of *Gdf15* ^fl/fl^ and *Gdf15* ^cKO^ mice. Single‐cell suspension preparation of subcutaneous tumor in *Gdf15* ^fl/fl^ and *Gdf15* ^cKO^ mice was described as above. The experiment was performed according to the product manual. The purity of the isolated cells was assessed by flow cytometry analysis (Figure S4A).

### 4.12 Phagocytosis Assay by Flow Cytometry

First, BMDMs from *Gdf15* ^fl/fl^ or *Gdf15* ^cKO^ mice and the GFP‐expressing MOC1 cells were both detached, counted, and incubated in a 96‐well U‐bottom ultra‐low attachment plate in a ratio of 1:10, 1:20, or 1:50 for 2 h. The cells were then put on ice to stop the phagocytosis and stained with PE‐F4/80 (565410, BD, USA) for flow cytometry analysis. The proportion of F4/80^+^ cells to GFP^+^ cells was calculated to represent the percentage of tumor cells phagocytosed by macrophages. Second, BMDMs from *Gdf15* ^fl/fl^ or *Gdf15* ^cKO^ mice were treated with 0.2 mg/mL of FITC‐OVA (SF069, Solarbio, China) for 15 min. The supernatant was then discarded and the BMDMs were rinsed with PBS 3 times to remove the FITC‐OVA adhered on the cell surface. The cells were then collected and stained with PE‐F4/80 (565410, BD, USA) for flow cytometry analysis. The uptake rate of FITC‐OVA was calculated as the proportion of FITC^+^ cells to F4/80^+^ cells. The MFI of FITC in F4/80^+^ cells was also calculated to represent the phagocytic ability. To explore the effect of exogenous GDF15 on phagocytosis, BMDMs from *Gdf15* ^fl/fl^ or *Gdf15* ^cKO^ mice were pretreated with 5 ng/mL of recombinant mouse GDF15 protein (10596‐GD, R&D Systems, USA) for 24 h, and FITC‐OVA was applied as described above in the presence of rGDF15 to assess the phagocytic ability.

### 4.13 Phagocytosis Assay by Confocal Microscopy

First, BMDMs (1×10^5^) from *Gdf15* ^fl/fl^ or *Gdf15* ^cKO^ mice were seeded in confocal dishes and cultured for 24 h. 0.2 mg/mL of FITC‐OVA (SF069, Solarbio, China) was then added and incubated for 15 min. The supernatant was then discarded and the BMDMs were rinsed with PBS 3 times to remove the FITC‐OVA absorbed on the cell surface. The cells were imaged by LSM 900 confocal laser microscopy (ZEISS, Germany). The integrated density of FITC was calculated via Fiji (version 2.9.0). Second, MOC1 cells (1 × 10^5^) were labeled with CFSE using CellTrace CFSE (C34554, ThermoFisher, USA) according to the manufacturer's instructions and cocultured with the same amount of BMDMs from *Gdf15* ^fl/fl^ or *Gdf15* ^cKO^ mice in confocal dishes for 24 h. Immunofluorescent staining of F4/80 was then performed on the cells as described below. The images were captured by LSM 900 confocal laser microscopy (ZEISS, Germany).

### 4.14 In Vivo Phagocytosis Detection Assay

MOC1‐GFP (1 × 10^6^) cells were inoculated subcutaneously into the right back of 6 to 8‐week‐old *Gdf15* ^fl/fl^ and *Gdf15* ^cKO^ mice. The single‐cell suspensions of the tumors were prepared and stained with APC‐Cy7‐CD45 (557659, BD, USA) and PE‐F4/80 (565410, BD, USA) for flow cytometry analysis. The proportion of GFP^+^ cells to F4/80^+^ cells was calculated to represent the phagocytic ability of TAMs. The MOC1 tumor without GFP expression was used as a negative control.

### 4.15 In Vitro Antigen Presentation Assay

First, BMDMs (1 × 10^6^) from *Gdf15* ^fl/fl^ or *Gdf15* ^cKO^ mice were seeded in 6‐well plates and treated with 20 µg/mL of SIINFEKL peptide (HY‐P1489, MCE, USA) for 2 h. The supernatant was then discarded and the cells were rinsed with PBS for 3 times. The cells were collected and stained with PE‐F4/80 (565410, BD, USA) and APC anti‐mouse H‐2Kb bound to SIINFEKL (141605, Biolegend, USA) for flow cytometry analysis. The antigen presentation ability was evaluated through the proportion of F4/80^+^H‐2Kb‐SIINFEKL^+^ cells. Second, BMDMs from *Gdf15* ^fl/fl^ or *Gdf15* ^cKO^ mice were treated with 20 µg/mL of SIINFEKL peptide (HY‐P1489, MCE, USA) for 6 h. The cells were then rinsed with PBS 3 times and cocultured with CD8^+^ T cells isolated from the spleens of OT‐1 mice and labeled with CFSE for 3 days. The ability of macrophages to stimulate CD8^+^ T cells proliferation was evaluated by flow cytometry. MojoSort mouse CD8^+^ T cells isolation kit (480008, Biolegend, USA) was used for the isolation of CD8^+^ T cells according to the manufacturer's instruction. CellTrace CFSE (C34554, ThermoFisher, USA) was used to label CD8^+^ T cells according to the manufacturer's instructions.

### 4.16 In Vivo Antigen Presentation Assay

The PGMLV‐CMV‐Chicken_OVA‐3 × Flag‐PGK‐Blasticidin lentivirus was constructed by Genomeditech Co., Ltd (Shanghai, China). MTCQ1 cells were transduced with the lentivirus for 48 h and then cultured with the medium containing 10 µg/mL of blasticidin for 7 days to establish the cell lines that stably express OVA. The expression of OVA‐Flag was verified by real‐time PCR and western blotting. Then, MTCQ1‐OVA cells (1×10^6^) were inoculated subcutaneously into the right back of 6‐8 weeks of *Gdf15* ^fl/fl^ and *Gdf15* ^cKO^ mice. The proportion of TAMs expressing H2Kb‐SIINFEKL was detected by flow cytometry to represent the antigen‐presenting ability.

### 4.17 Apoptosis Assay

BMDMs (5 × 10^5^) from *Gdf15* ^fl/fl^ or *Gdf15* ^cKO^ mice were cocultured with the same amount of MOC1 cells in 6‐well plates for 24 h. The cells were detached using scrapers and centrifuged together with the supernatant at 500 g for 5 min. The cells were first stained with APC‐Cy7‐CD45 (557659, BD, USA). The apoptosis indicators were then stained using an Annexin V‐FITC apoptosis detection kit (C1062S, Beyotime, China). The apoptosis rate of MOC1 cells (CD45^−^ cells) was evaluated by flow cytometry.

### 4.18 Polarization of BMDMs

BMDMs (1 × 10^6^) from *Gdf15* ^fl/fl^ or *Gdf15* ^cKO^ mice were seeded in 6‐well plates and cultured for 24 h. The BMDMs were then stimulated for 24 h with 20 ng/mL of interferon‐γ (IFN‐γ, 50709‐MNAH, SinoBiological, China) and 100 ng/mL of lipopolysaccharide (LPS, L2880, Sigma–Aldrich, USA) for M1 polarization or 20 ng/mL of interleukin‐4 (IL‐4, 51084‐MNAE, SinoBiological, China) for M2 polarization.

### 4.19 CRISPR‐Cas9‐Mediated Knockout in THP‐1 Cells

The *GDF15* knockout THP‐1 cell line was constructed by Genomeditech Co., Ltd (Shanghai, China). Briefly, Cas9 and sgRNA were transfected into THP‐1 cells by electroporation. The DNA was extracted, amplified by PCR, and then sequenced for verification after 72 h. The primers were as follows: forward 5’‐ GCTGTGGTCATTGGAGTGTTTAC‐3’ and reverse 5’‐ GCTTTTCAAGATGAGGAATCCC‐3’. Monoclonal cultivation was then performed on the polyclonal *GDF15* knock‐out cells. Each monoclonal cell went through culture expansion, DNA extraction, PCR amplification and sequencing, and the monoclonal *GDF15* knock‐out THP‐1 cell line was finally established.

### 4.20 GDF15 Nuclear Localization Detection

First, western blotting was performed on the nucleoprotein of RAW 264.7 cells extracted using a nuclear protein extraction kit (P0027, Beyotime, China) to detect the nuclear distribution of GDF15. Second, RAW 264.7 cells were seeded in confocal dishes, and immunofluorescent staining of GDF15 and DAPI was then performed on the cells as described below to observe the nuclear distribution of GDF15. The images were captured by LSM 900 confocal laser microscopy (ZEISS, Germany).

### 4.21 Elisa

BMDMs (8 × 10^5^) from *Gdf15* ^fl/fl^ or *Gdf15* ^cKO^ mice were seeded in 6‐well plates and cultured for 48 h with 1.5 mL of culture medium in each well. The culture supernatant was collected and centrifuged at 2000 RPM for 10 min. The concentration of GDF15 in the supernatant was measured using a Mouse/Rat GDF15 Quantikine ELISA Kit (MGD150, R&D Systems, USA). The absorbance was measured by SpectraMax M3 (Molecular Devices, USA). For each genotype, three mice were sacrificed for the isolation of BMDMs. Each sample was measured in technical duplicates.

### 4.22 Immunofluorescence

The cells were fixed with 4% paraformaldehyde for 10 min, treated with 0.1% Triton X‐100 for 5 min, and blocked with QuickBlock blocking buffer (P0260, Beyotime, China) for 10 min. The cells were incubated with primary antibodies and then fluorescent secondary antibodies for 1 h each at room temperature. As for primary antibodies, F4/80 antibody (1:400, 28463‐1‐AP, Proteintech, China), GDF15 (10 µg/mL, AF6385, R&D Systems, USA), phospho‐NF‐κB p65 (Ser536) (1:1000, 3033, Cell Signaling Technology, USA) were used for immunofluorescence. As for secondary antibodies, anti‐rabbit IgG (H + L)‐F(ab')2 fragment (Alexa Fluor 594 conjugate) (1:500, 8889, Cell Signaling Technology, USA) and anti‐sheep IgG (H + L)‐FITC (1:200, BS30950, Bioworld Technology, USA) were used for immunofluorescence. DAPI Staining Solution (C1006, Beyotime, China) was used for nuclear staining.

### 4.23 Real‐Time PCR

The total RNA of the cultured cells was extracted using RNA‐Quick Purification Kit (RN001, ES Science, China). The concentration and purification of RNA was assessed by NanoDrop 2000 (Thermo Scientific, USA). PrimeScriptRT master mix (RR036, Takara, Japan) was used to reverse RNA into cDNA. Then TB Green premix ex Taq (RR420, Takara, Japan) was used to perform real‐time PCR on the StepOnePlus real‐time PCR system (Applied Biosystems, USA). The primers were synthesized by Sangon Biotech (Shanghai, China). All the primers used for real‐time PCR are listed in Table S1.

### 4.24 Western Blotting

The cell lysates were separated by SDS‐PAGE and transformed to a 0.22 µm PVDF membrane. The membrane was blocked for 1 h at room temperature with 5% BSA in TBST and then incubated with primary antibody overnight at 4°C. The membrane was incubated with the corresponding secondary antibodies conjugated with HRP, developed with high‐sig ECL (180‐501, Tanon), and scanned by Amersham Imager 600. The primary antibodies were as follows: β‐ACTIN (1:1000, 5125, Cell Signaling Technology), GAPDH (1:50000, 60004‐1‐Ig, Proteintech), NF‐κB p65 (1:1000, 8242, Cell Signaling Technology), phospho‐NF‐κB p65 (Ser536) (1:1000, 3033, Cell Signaling Technology), IκBα (1:1000, 9242, Cell Signaling Technology), phospho‐IκBα (Ser32) (1:2000, ab92700, Abcam), Complement 3 (1:5000, 21337‐1‐AP, Proteintech), GDF15 (1:1000, NBP2‐44214, NOVUS), Flag (1:1000, GM‐30726AB, Genomeditech), Lamin B1 (1:1000, ab16048, Abcam).

### 4.25 RNA‐Seq and Analysis

The isolation of TAMs from subcutaneous tumors of *Gdf15* ^fl/fl^ and *Gdf15* ^cKO^ mice was described above. The isolated cells were then resuspended in TRIzol (T9424, Sigma–Aldrich, USA) to extract total RNA. The RNA‐seq was performed by Cosmos Wisdom Co., Ltd (Hangzhou, China). The Illumina Novaseq 6000 platform was used for sequencing. Illumina TruSeq RNA sample prep Kit was used for library construction. The gene expression level was calculated using FPKM. *p* value <0.05 and |fold change| >1.5 were set as the threshold for DEGs. Further Gene Ontology (GO) and Kyoto Encyclopedia of Genes and Genomes (KEGG) enrichment analysis were performed with the DEGs to illustrate the biological functions and pathway differences between the *Gdf15* ^fl/fl^ and *Gdf15* ^KO^ TAMs.

### 4.26 ChIP‐Seq

THP‐1 cells were seeded in a 15 cm culture dish and treated with 100 ng/mL of Phorbol 12‐myristate 13‐acetate (PMA, P1585, Sigma Aldrich, USA) for 24 h to induce into macrophages. A ChIP assay was performed using a SimpleChIP Enzymatic Chromatin IP Kit (9003, Cell Signaling Technology, USA) according to the manufacturer's instructions. GDF15 antibody (1:30, ab206414, Abcam, USA) was used for ChIP. The sequencing was performed by Cloud‐Seq Co., Ltd (Shanghai, China). Briefly, the sequencing libraries were generated with GenSeq Rapid DNA Library Prep Kit (Cloud‐Seq Co., Ltd) according to the manufacturer's instruction. The library quality was determined by using Agilent 2100 Bioanalyzer (Agilent, China), and then subjected to high‐throughput 150 base paired‐end sequencing on Illumina NovaSeq sequencer. Peak calling was performed with MACS software (version 1.4.2). The enriched peaks were then annotated with the latest UCSC RefSeq database to connect the peak information with the gene.

### 4.27 Flow Cytometry

Single‐cell suspension preparation was described as above. The staining process was performed according to the protocols provided by Biolegend (https://www.biolegend.com/ ). Briefly, red blood cells in single cell suspensions were lysed using RBC lysis buffer (420301, Biolegend, USA). Fixable viability stain 510 (FVS510, 564406, BD, USA) was used to identify live cells. TruStain FcX PLUS (156603, Biolegend, USA) was used for Fc blockade. Cell surface staining was performed on ice for 30 min. Cells were then fixed with 4% paraformaldehyde for 20 min at room temperature, followed by permeabilization using intracellular staining permeabilization wash buffer (421002, BioLegend, USA). Intracellular staining was performed on ice overnight.

As for the staining of GDF15^+^ TAMs in human OSCC, the single cell suspension after RBC lysis was treated with Brefeldin A (1:1000, 420601, Biolegend, USA) at 37°C for 6 h in RPMI 1640 culture medium to block the secretion of GDF15. FVS510 staining, cell surface staining, fixation, and permeabilization were performed in sequence as stated above. Avidin/Biotin Blocking kit (ab64212, Abcam, USA) was used to block endogenous biotin. Intracellular staining of GDF15 (biotinylated) and CD68 was performed, followed by the staining of streptavidin‐PE (0.06 µg/test, 12‐4317‐87, Invitrogen, USA) on ice for 30 min.

As for the staining of H2Kb/β2M/OVA (SIINFEKL) tetramer, the tetramer was added (0.25 µg/test) right after Fc blockade and treated at 4°C for 60 min. FVS510 staining and cell surface staining were performed in sequence afterward.

The following antibodies were used in this study: APC‐Cy7‐CD45 (557659, BD), APC‐CD3e (553066, BD), PerCP‐Cy5.5‐CD4 (550954, BD), FITC‐CD8a (553030, BD), APC‐H7‐CD8a (560182, BD), PE‐F4/80 (565410, BD), BV421‐CD86 (564198, BD), PE‐Cy7‐CD163 (25‐1631‐82, Invitrogen, USA), BV421‐CD279 (566878, BD), PE‐TIM3 (566346, BD), PE‐Cy7‐Granzyme B (372213, Biolegend), APC‐CD274 (564715, BD), APC anti‐mouse H‐2Kb bound to SIINFEKL (141605, Biolegend), PE‐Cy7‐CD25 (102015, Biolegend), PerCP‐Cy5.5‐CD69 (104521, Biolegend), PE‐labeled mouse H2Kb/β2M/OVA (SIINFEKL) tetramer (H2A‐MP2H7, Acro Biosystems), AF647‐CD68 (353015, Biolegend), biotinylated‐GDF15 (1:100, biotinylated, TA421293AM, OriGene, USA). Flow cytometry was performed on BD FACSCanto II (Becton, Dickinson and Company, USA) and NovoCyte Peatean (Agilent, China). Data were analyzed using FlowJo 10.8.1 and NovoExpress software. The gating strategies were illustrated in Figures S2D and S3E.

### 4.28 mIHC Staining

The specimens were fixed in 4% paraformaldehyde for 48 h, embedded in paraffin, and sectioned with a thickness of 4 µm. The sections were heated at 65°C for 30 min. mIHC staining was then performed using a 7‐color IHC kit (abs50015, Absin, China) according to the manufacturer's instructions. The primary antibodies used for mIHC staining were as follows: NK1.1 (1:100, 39197, Cell Signaling Technology), CD19 (1:6400, 90176, Cell Signaling Technology), F4/80 (1:8000, 28463‐1‐AP, Proteintech), CD4 (1:400, 25229, Cell Signaling Technology), CD8 (1:2000, ab217344, Abcam).

### 4.29 scRNA‐seq Analysis

All the analyses were performed in the R 4.3.1 environment. Raw count matrices were processed using the Seurat R package (v4.3.0) with stringent quality control thresholds: only high‐quality cells retaining more than 300 unique genes (nFeature_RNA), more than 1000 total UMIs (nCount_RNA), and less than 20% mitochondrial gene content were retained. The data were then normalized using the LogNormalize method with a scale factor of 10 000. The top 2000 highly variable genes were identified, and scaling and PCA dimensionality reduction were performed. To mitigate the impact of doublets on downstream analyses, the DoubletFinder tool (with parameters pN = 0.25, pK = 0.09) was employed to predict and remove doublet cells, resulting in high‐quality single‐cell data for subsequent analysis. To correct for technically driven batch effects while preserving biologically meaningful variation, data integration was carried out using the Harmony algorithm (v1.21). Differential expression analysis for each cell subpopulation was conducted using the MAST test to identify marker genes for cell type annotation. Expression patterns of *GDF15* were visualized using VlnPlot and DotPlot. Macrophages were subsequently extracted and re‐analyzed separately.

### 4.30 Statistical Analysis

GraphPad Prism (Version 9) was used to process the initial data and perform statistical analysis. Data were assumed to follow a normal distribution in all in vivo and in vitro experiments in this study, and parametric testing methods were used for analysis. Due to the low power of the normality test in analyses with small sample sizes, the normality test was not formally performed. Comparisons between two groups were performed using a two‐tailed unpaired Student's *t*‐test. One‐way ANOVA (three or more groups), or two‐way ANOVA (two independent variables influencing a continuous dependent variable) was applied as appropriate, followed by Tukey's multiple comparisons test. *p* < 0.05 was considered statistically significant. All data were presented as mean ± standard deviation (SD).

## Author Contributions

X.Y.Z. and Z.H.Z. designed and conducted most of the in vitro and in vivo experiments. H.Y.J. collected the OSCC samples and supervised the sequencing and analysis of scRNA‐seq. C.Y.G. conducted scRNA‐seq analysis. Z.H.Z. and T.C.Z. supervised the construction of Gdf15^cKO^ mice. F.X.Z. performed the ChIP experiment and helped with mIHC staining and in vivo experiments. Y.Y.Z. helped with scRNA‐seq analysis and in vivo experiments. X.C. helped with flow cytometry analysis. X.Y.Z. was responsible for acquiring and analyzing data. X.Y.Z. wrote the original manuscript. J.Z.H., L.P.Z., Z.H.Z. and T.C.Z. reviewed and edited the manuscript.

## Funding

This work was financially supported, in whole or in part, by the National Natural Science Foundation of China (82473268; 82172734; 82372623), Excellent Academic Leader Program of Shanghai Oriental Talent Plan (BJWS2024045), Clinical Scientist Development Program of Fudan University (DGF828024‐2/032), Shanghai Sailing Program grant (22YF1421700; 24YF2722800). The funding source had no role in the design of the study, collection, analysis, and interpretation of data and in writing the manuscript.

## Ethics Statement

All the laboratory procedures concerning mice were approved by the laboratory animal care and use committee of Ninth People's Hospital, Shanghai Jiao Tong University, China (SH9H‐2024‐A418‐SB). All the procedures concerning human samples were approved by the ethics committee of Ninth People's Hospital, Shanghai Jiao Tong University, China (SH9H‐2020‐T382‐9).

## Conflicts of Interest

The authors declare no conflicts of interest.

## Supporting information




**Supporting File**: advs74796‐sup‐0001‐SuppMat.docx.

## Data Availability

The RNA‐seq and ChIP‐seq data generated in this study were deposited in the Sequence Read Archive (SRA) database with accession numbers PRJNA1329226 and PRJNA1329947. The remaining data that support our study are available from the corresponding authors upon request.
