# Supplementary material for: Context‐Dependent Role of GDF15: GDF15+ Tumor‐Associated Macrophages Suppress OSCC Progression by Enhancing Phagocytosis
Source: Adv Sci (Weinh). 2026 Mar 13;13(29):e18525. doi: 10.1002/advs.202518525 (PMC13205754; doi:10.1002/advs.202518525)
Supplement: Supplementary file 1 — Supporting File: advs74796‐sup‐0001‐SuppMat.docx. [file ADVS-13-e18525-s001.docx]

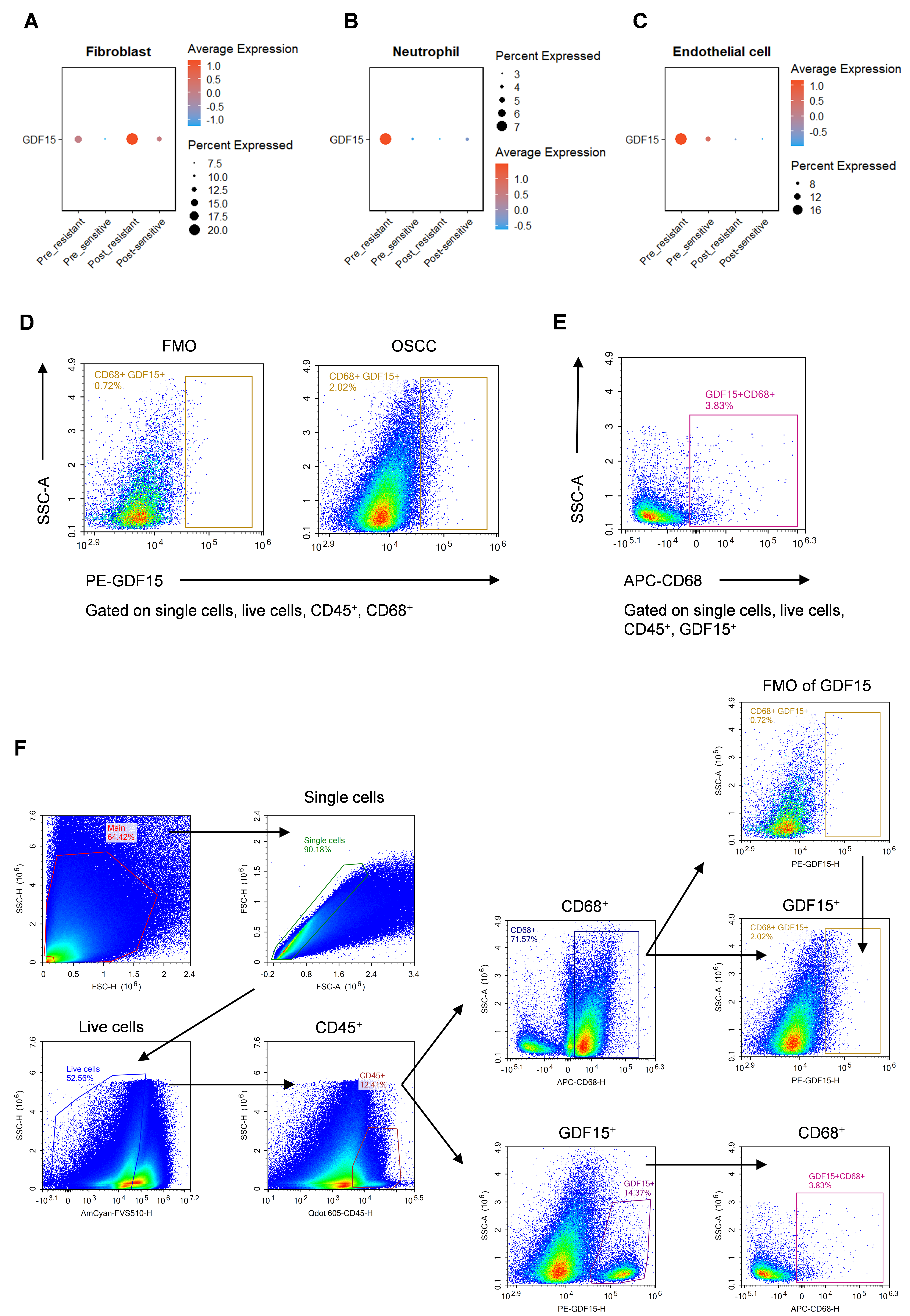
Supplementary figures

Supplementary figure 1. Expression of GDF15 in OSCC samples pre and post ICB-based treatment. (A-C) Dot plots comparing the expression levels of *GDF15* in the four groups (pre-resistant, pre-sensitive, post-resistant, post-sensitive) in fibroblasts (A), neutrophils (B) and endothelial cells (C) in OSCC tumor tissues. (D-E) Flow cytometry verification of GDF15^+^ TAMs in human OSCC, showing the proportion of GDF15^+^ cells in CD68^+^ TAMs (D) and the proportion of CD68^+^ TAMs in GDF15^+^ immune cells (E). (F) Gate strategy of GDF15^+^ TAMs in human OSCC.


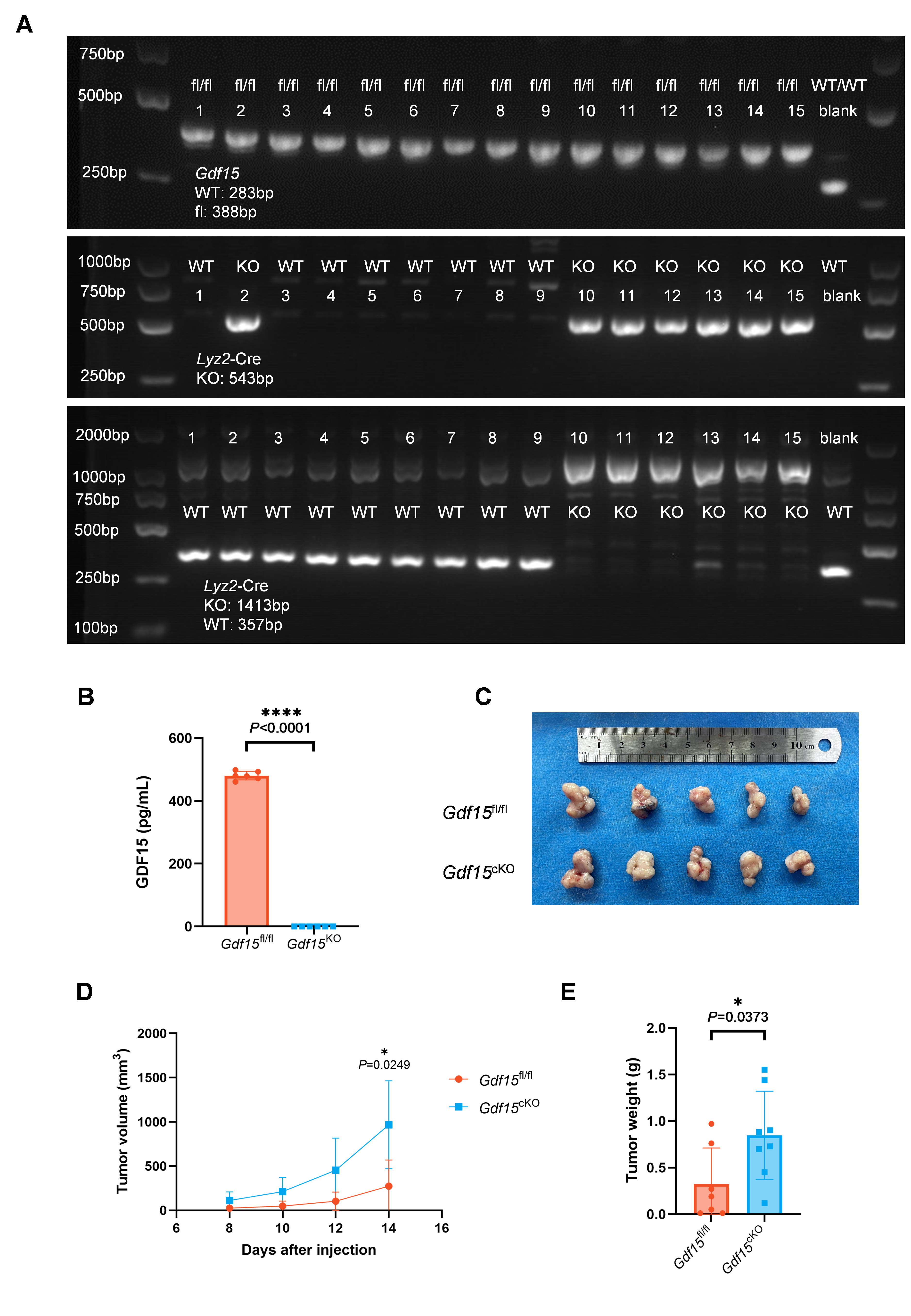


Supplementary figure 1.


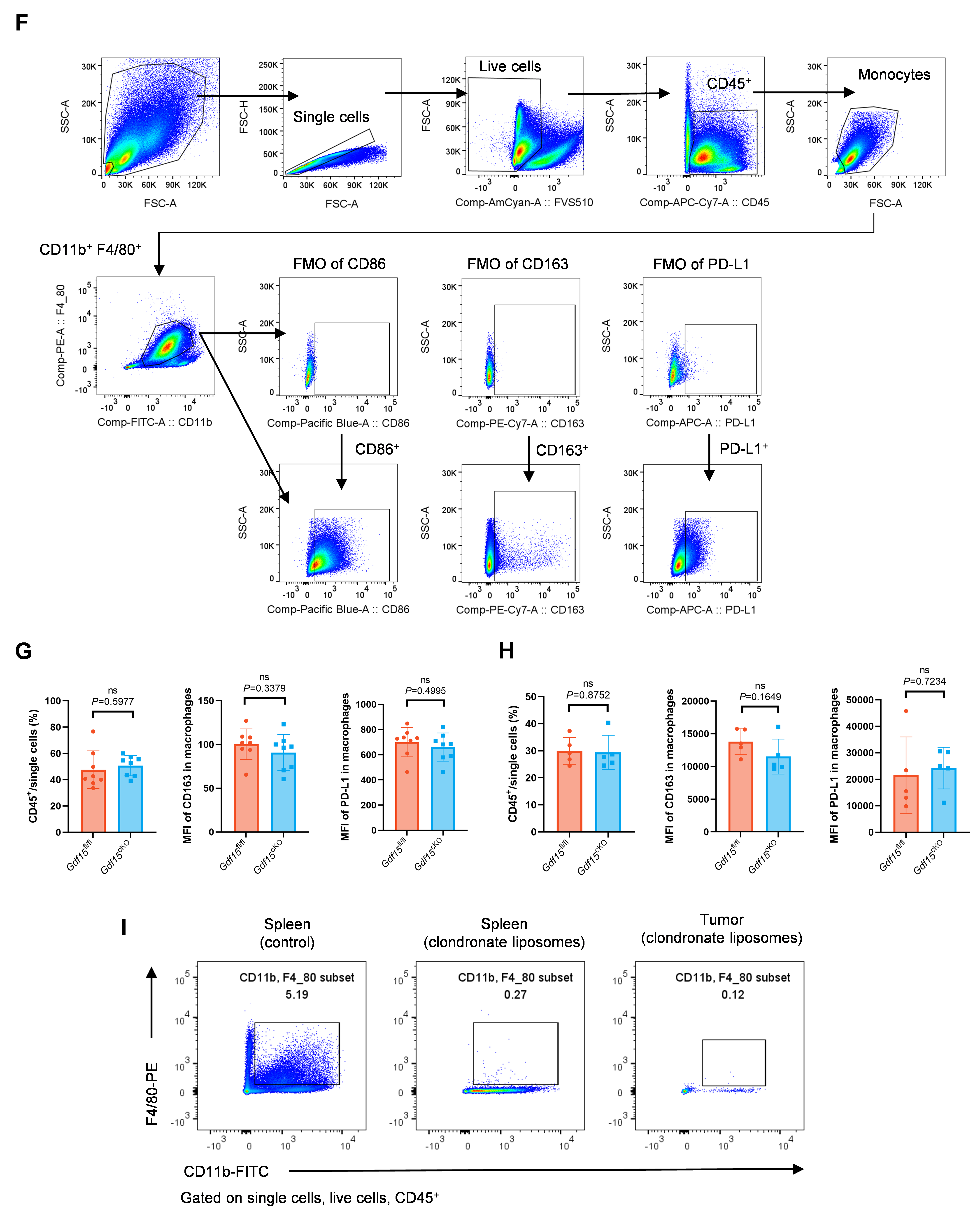


Supplementary figure 2. GDF15 deficiency in TAMs accelerates tumor growth. (A) Representative images showing the identification of *Gdf15*^fl/fl^ and *Gdf15*^cKO^ mice genotypes. (B) Concentration of GDF15 in the culture supernatant of *Gdf15*^fl/fl^ and *Gdf15*^KO^ BMDMs. (C) Image of tumors dissected from MOC1-bearing *Gdf15*^fl/fl^ and *Gdf15*^cKO^ mice (*n*=5). (D-E) Tumor volume curves (D) and tumor weight (E) of the B16 tumors in *Gdf15*^fl/fl^ and *Gdf15*^cKO^ mice. (F) The gate strategy of TAMs. (G-H) Statistical analysis of the percentage of CD45^+^ cells and the expression of CD163 and PD-L1 in macrophages in the TME of MTCQ1 (G) and MOC1 (H) tumors. (I) Flow cytometry verification of the efficiency of clodronate liposomes in systemic macrophage clearance. Statistical significance was analyzed via two-way ANOVA (D) and unpaired Student’s t-test (E, G, H). **P* < 0.05, *****P* < 0.0001. Data were presented as mean ± SD.


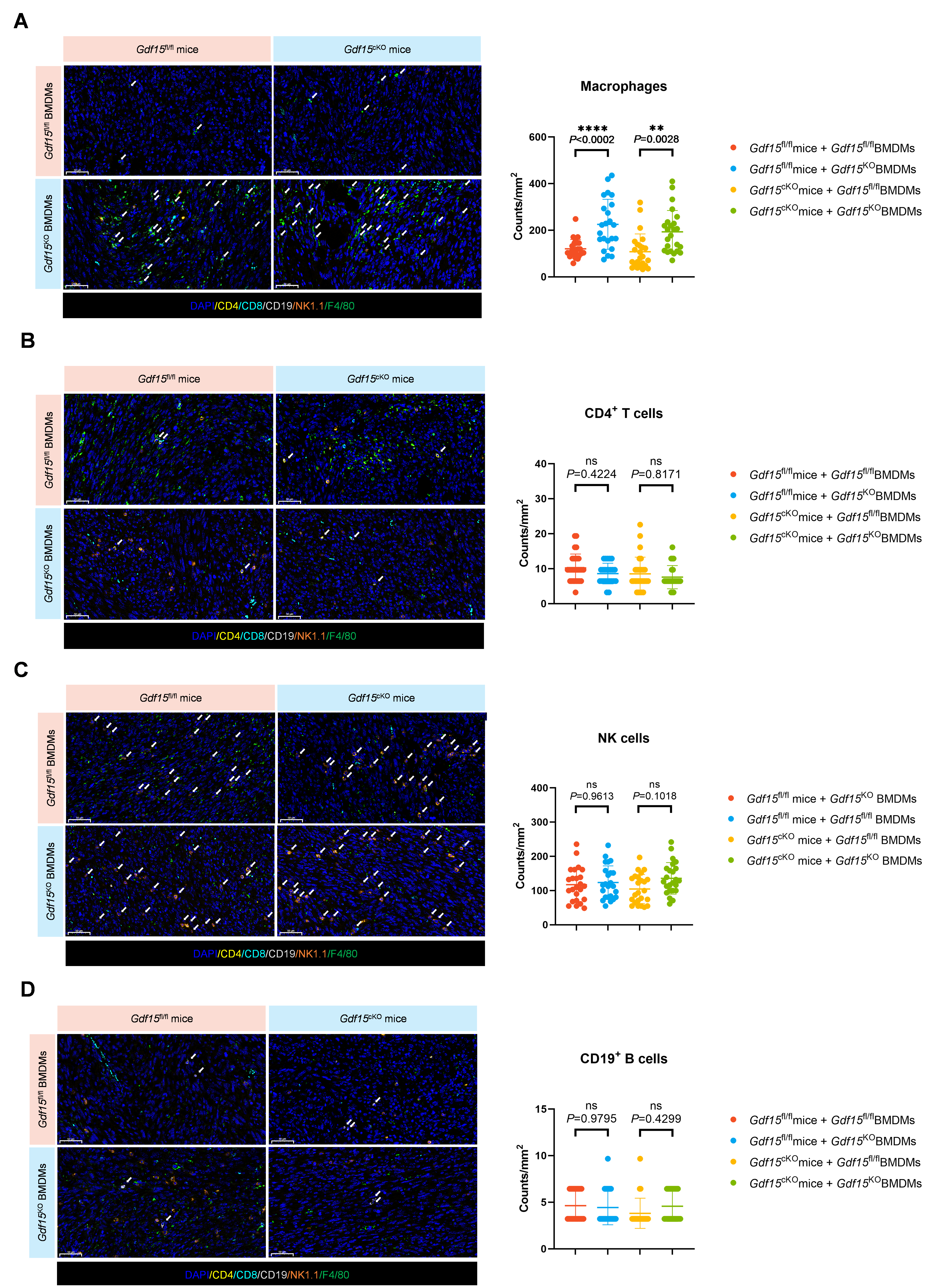


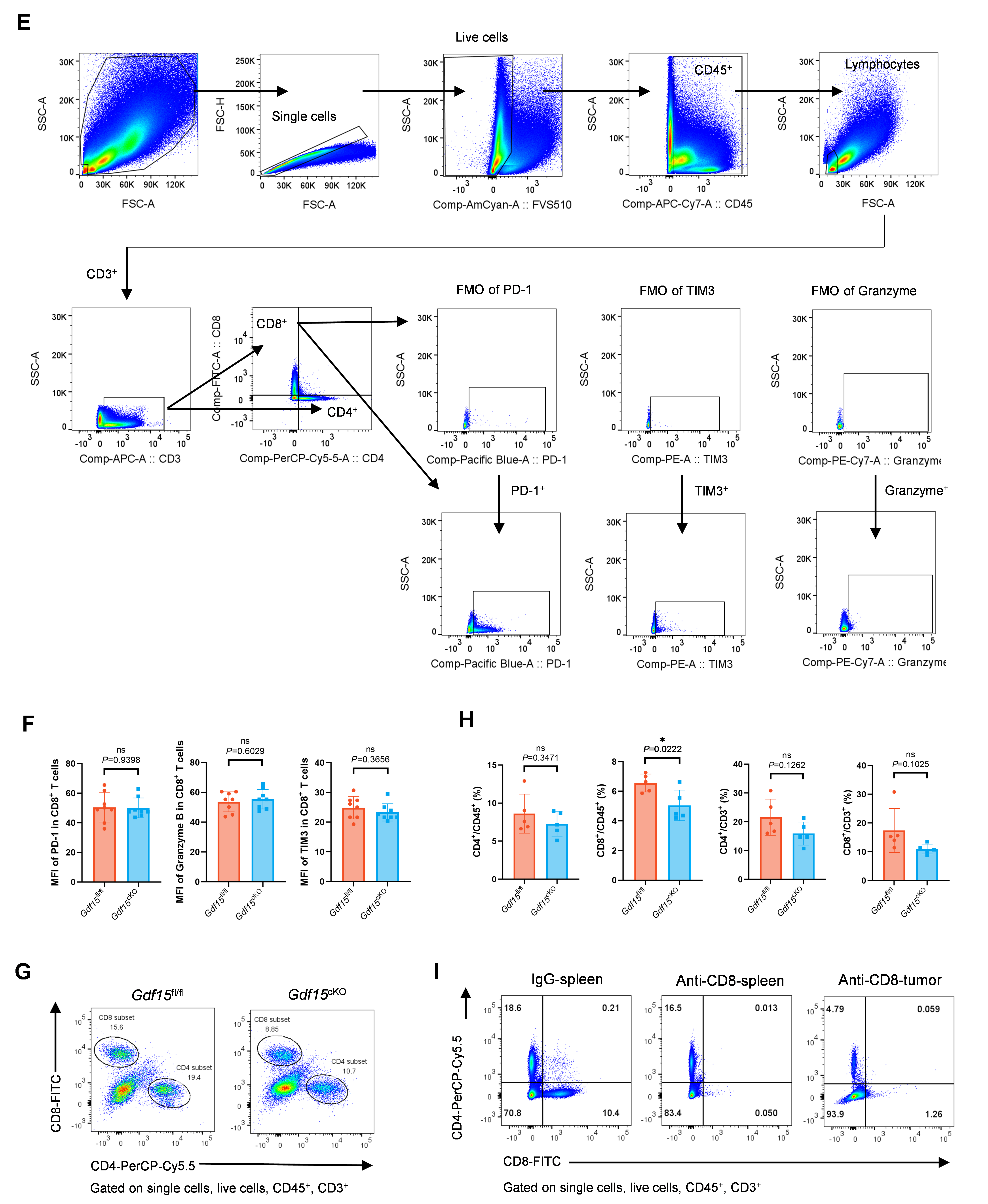


Supplementary figure 3. GDF15 deficiency in TAMs suppresses tumor infiltration of CD8^+^ T cells. (A-D) Representative mIHC staining images and statistical analysis of the density of F4/80^+^ macrophages (A), CD4^+^ T cells (B), NK cells (C), CD19^+^ B cells (D) in the TME of the tumor samples in Figure 2L. The white arrows pointed out the tumor-infiltrating F4/80^+^ macrophages (A), CD4^+^ T cells (B), NK cells (C), CD19^+^ B cells (D). Scale bar, 50 μm. (E) The gate strategy of tumor-infiltrating T cells. (F) Statistical analysis showing the expression of PD-1, Granzyme B and TIM3 in the TME of MTCQ1 tumors in *Gdf15*^fl/fl^ and *Gdf15*^cKO^ mice detected by flow cytometry. (G-H) Representative plots and statistical analysis showing the percentage of CD4^+^ and CD8^+^ T cells in the lungs of *Gdf15*^fl/fl^ and *Gdf15*^cKO^ mice i.v. injected with B16 cells (*n*=5). (I) Flow cytometry verification of the efficiency of CD8 antibody in the systemic blockade of CD8^+^ T cells. Statistical significance was analyzed via one-way ANOVA (A-D) and unpaired Student’s t-test (F, H). **P* < 0.05, ***P* < 0.01, *****P* < 0.0001. Data were presented as mean ± SD.


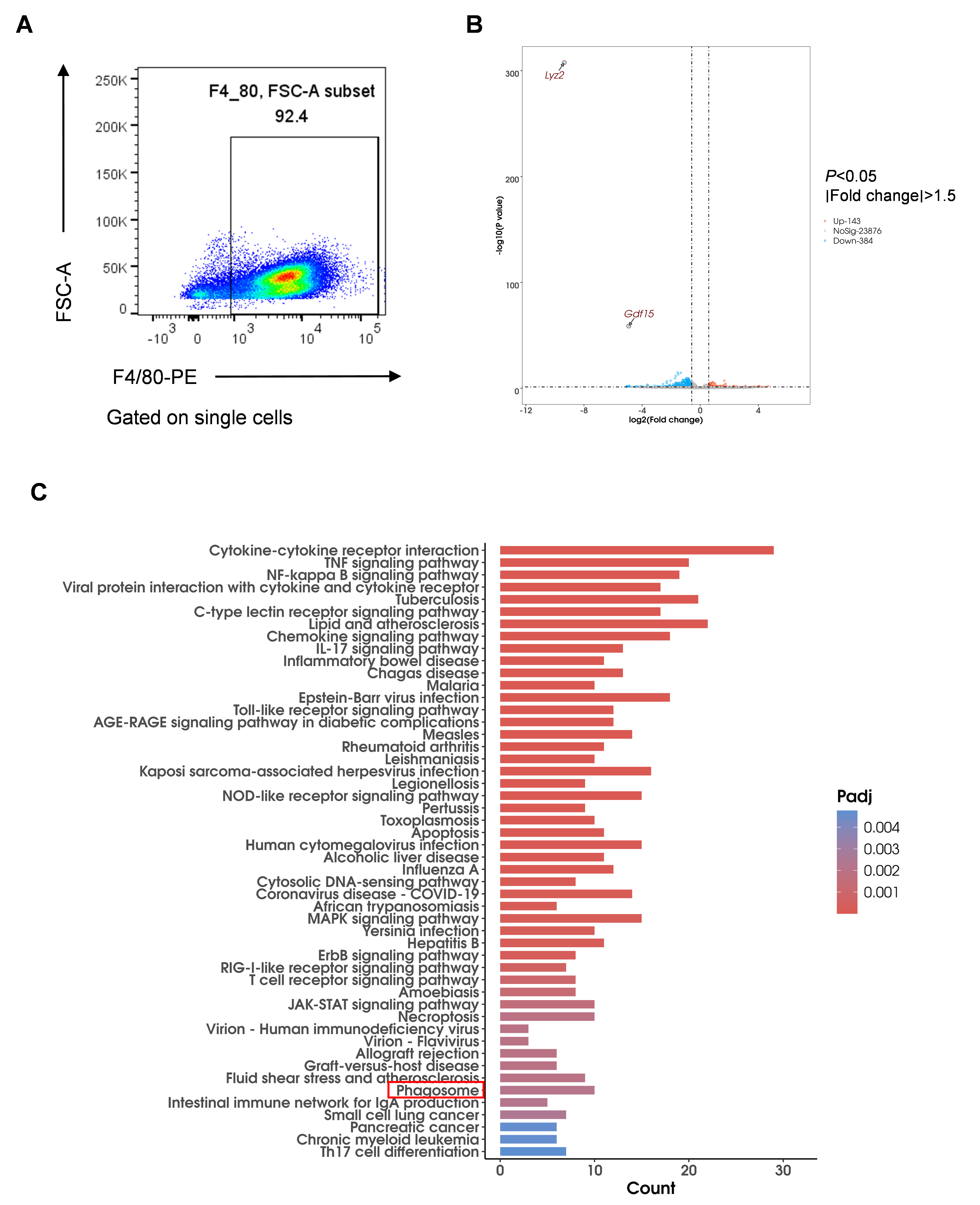


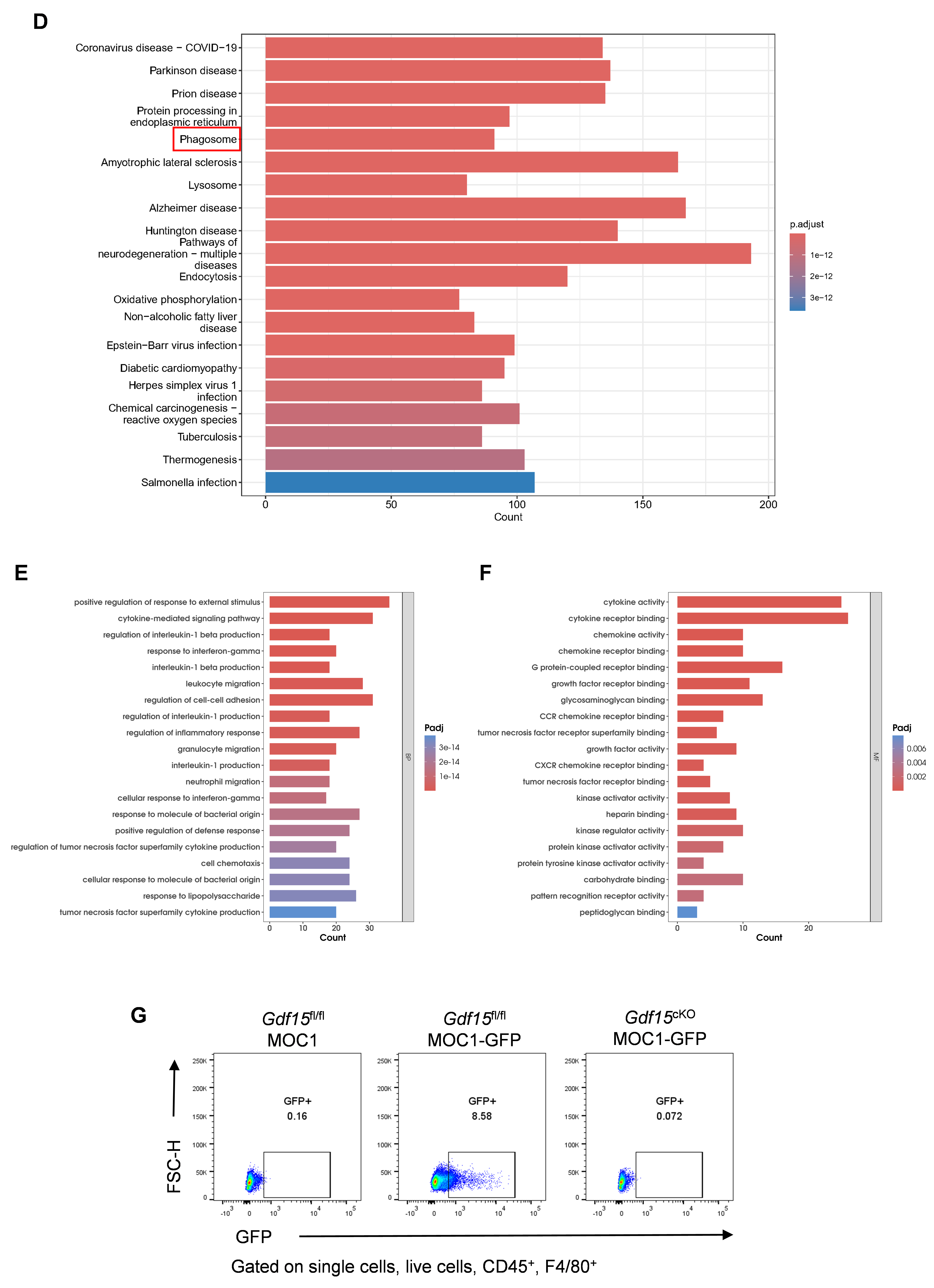


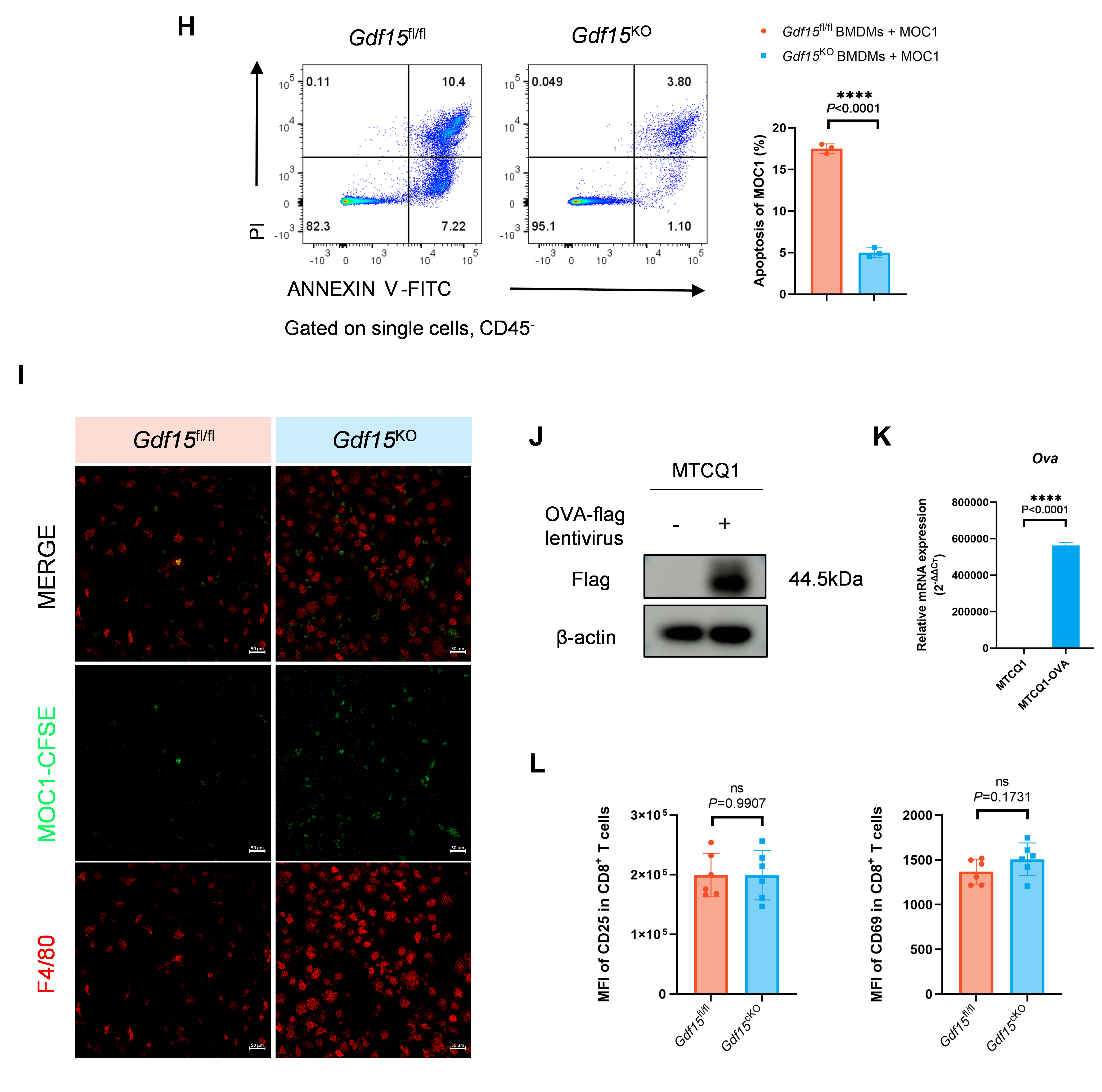
Supplementary figure 4. GDF15 enhances phagocytosis and antigen cross-presentation of macrophages. (A) Flow cytometry verification of the efficiency of F4/80 magnetic beads in the isolation of TAMs. (B) Volcano plot of the DEGs between *Gdf15*^fl/fl^ and *Gdf15*^KO^ TAMs. The extremely down-regulated expression of *Gdf15* and *Lyz2* was pointed out to verify the knock-out efficacy. (C) Bar plot showing the top 50 down-regulated pathways enriched by KEGG analysis in *Gdf15*^KO^ TAMs. Phagosome pathway was marked out with a red frame. (D) Bar plot showing the top 20 upregulated pathways in *GDF15*^+^ TAMs versus *GDF15*^-^ TAMs enriched by KEGG analysis. (E-F) Bar plot showing the top 20 down-regulated terms enriched by GO analysis of BP (C) and MF (D) in *Gdf15*^KO^ TAMs. (G) Representative plots showing the MOC1-GFP cells recently being phagocytosed by TAMs in *Gdf15*^fl/fl^ and *Gdf15*^cKO^ mice detected by flow cytometry. (H) Apoptosis assay of MOC1 cells when co-cultured with *Gdf15^f^*^l/fl^ or *Gdf15*^KO^ BMDMs. (I) Representative confocal images of MOC1-CFSE co-cultured with *Gdf15*^fl/fl^ or *Gdf15*^KO^ BMDMs. Scale bar, 50 μm. (J-K) Western blotting and real-time PCR verification of the expression of OVA-flag in the MTCQ1 cells transfected with lentivirus. (L) Statistical analysis of the expression of CD25 and CD69 in CD8^+^T cells in subcutaneous MTCQ1-OVA tumors in *Gdf15*^fl/fl^ and *Gdf15*^cKO^ mice (*n*=6). Statistical significance was analyzed via unpaired Student’s t-test. *****P* < 0.0001. Data were presented as mean ± SD.


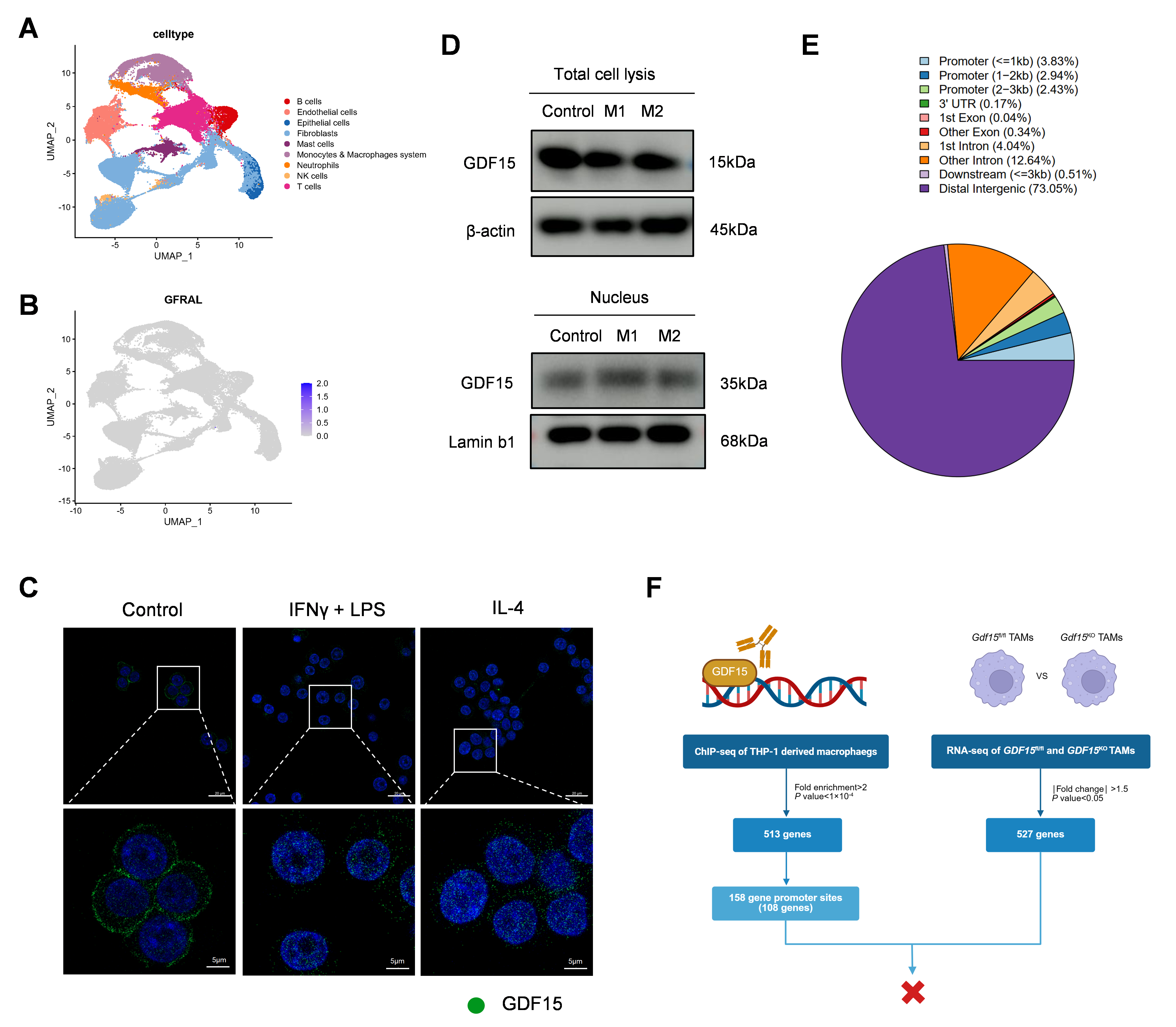
Supplementary figure 5. GDF15 could locate in the nucleus of macrophages but does not regulate the functions in the form of a transcription factor. (A-B) UMAP plot and feature plot showing the expression of GFRAL in OSCC tissues. (C) Representative confocal images showing the distribution of GDF15 within RAW 264.7 cells. Scale bar, 20 μm (the upper three images). Scale bar, 5 μm (the lower three images). (D) Western blotting analysis of GDF15 in the total cell lysis and the nuclear protein of RAW 264.7 cells. (E) Pie chart showing the distribution of GDF15-binding regions in the genome based on the results of ChIP-seq. (F) Schematic diagram showing the intersection process of the results from ChIP-seq in THP-1 derived macrophages and RNA-seq in TAMs.


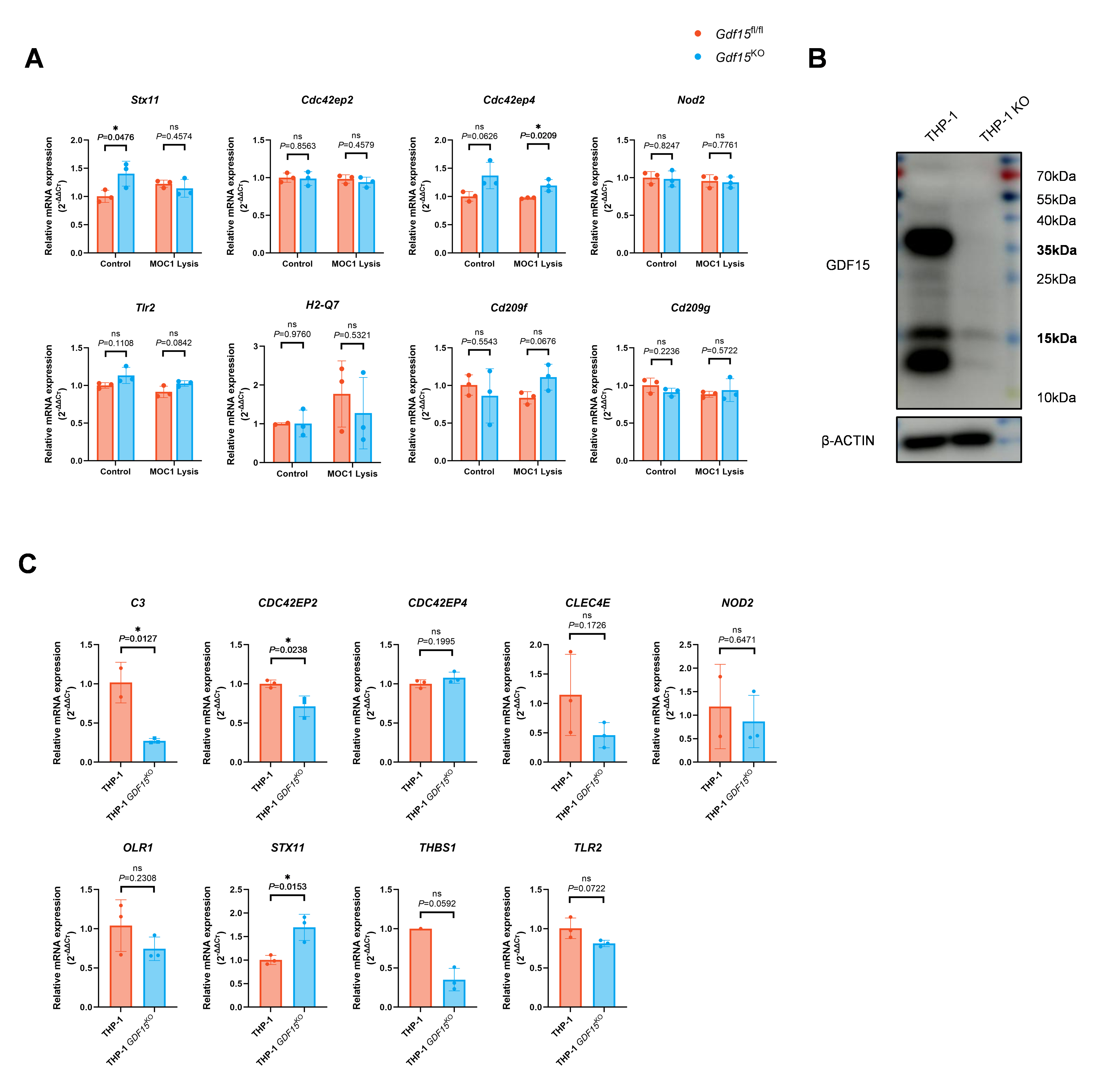
Supplementary figure 6. GDF15 regulates macrophage functions through the NF-κB pathway. (A) Real-time PCR verification in *Gdf15*^fl/fl^ and *Gdf15*^KO^ BMDMs of the phagocytosis or antigen-presentation related down-regulated genes in *Gdf15*^KO^ TAMs screened out by RNA-seq. (B) Western blotting verification of the *GDF15* knock-out efficacy in THP-1 derived macrophages. (C) Real-time PCR verification in macrophages derived from THP-1 cells and THP-1 *GDF15*^KO^ cells of the phagocytosis or antigen-presentation related down-regulated genes in *Gdf15*^KO^ TAMs screened out by RNA-seq. Statistical significance was analyzed via unpaired Student’s t-test. **P* < 0.05. Data were presented as mean ± SD.


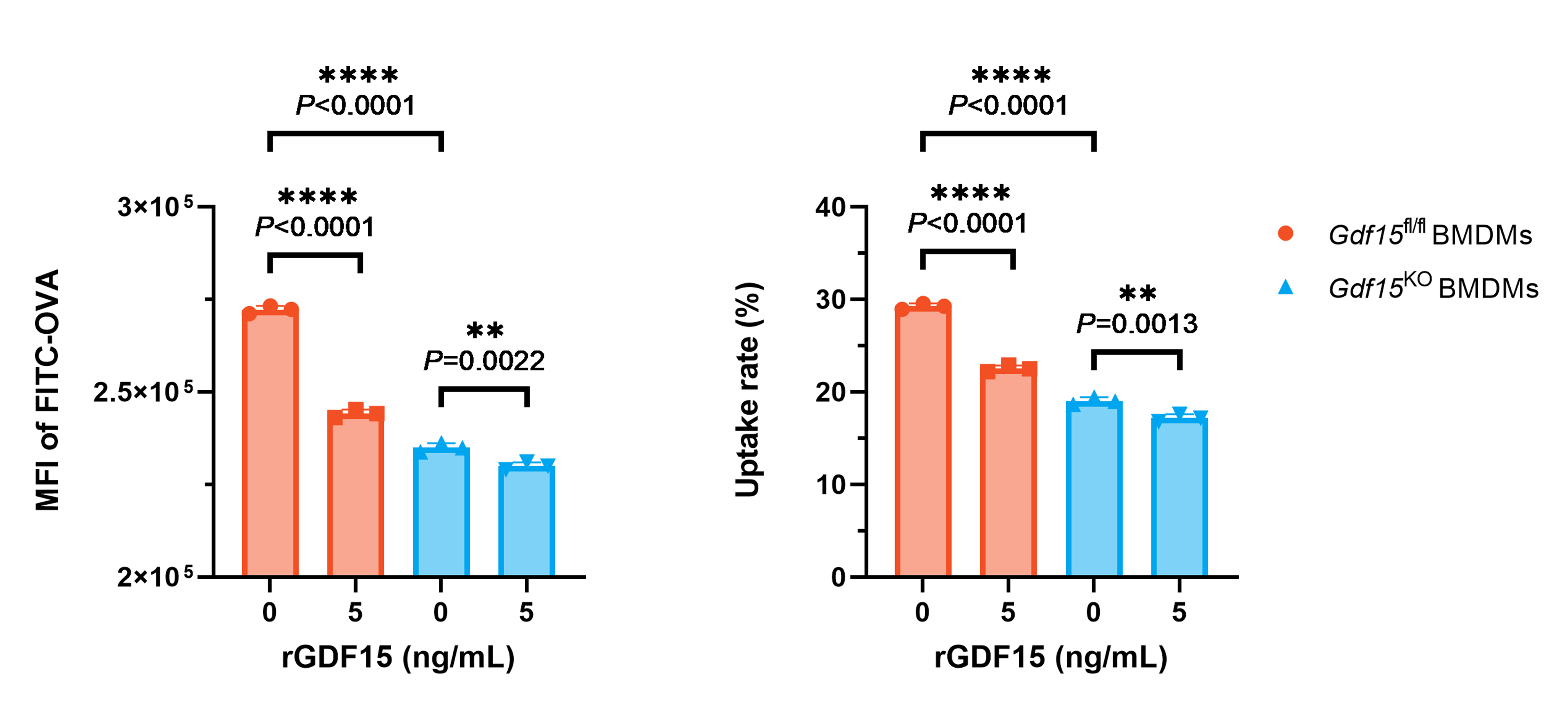
Supplementary figure 7. Recombinant GDF15 (rGDF15) could reduce phagocytosis of BMDMs. Statistical significance was analyzed via one-way ANOVA. ***P* < 0.01, *****P* < 0.0001. Data were presented as mean ± SD.

Supplementary table 1. Primers for real-time PCR

| Gene | Species | Forward primer | Reverse primer |
| --- | --- | --- | --- |
| *Tnfα* | Mouse | GTAGCCCACGTCGTAGCAAA | ACAAGGTACAACCCATCGGC |
| *Il6* | Mouse | TAGTCCTTCCTACCCCAATTTCC | TTGGTCCTTAGCCACTCCTTC |
| *Il12* | Mouse | TCTAGATGCTGGCCAGTACACC | ATTTGGTGCTTCACACTTCAGG |
| *Ccl2* | Mouse | TGTAGTTTTTGTCACCAAGCTCA | GTGCTTGAGGTGGTTGTGGA |
| *Ccl3* | Mouse | GCCAGGTGTCATTTTCCTGAC | TCGATGTGGCTACTTGGCAG |
| *C3* | Mouse | CCAGCTCCCCATTAGCTCTG | GCACTTGCCTCTTTAGGAAGTC |
| *Thbs1* | Mouse | GGGGAGATAACGGTGTGTTTG | CGGGGATCAGGTTGGCATT |
| *Clec4e* | Mouse | AGTGCTCTCCTGGACGATAG | CCTGATGCCTCACTGTAGCAG |
| *Stx11* | Mouse | TGTCCAGGAGCTATGACCAG | GTTGGTGTCGCGCTTAATGC |
| *Cdc42ep2* | Mouse | TCCCCATCTATTTGAAACGTGG | CCGCTGTTCCTGGAAGGAG |
| *Cdc42ep4* | Mouse | CAGCTCTGTGAACTCGAAGC | GCTAGTGAGGAAAGACGTGTCC |
| *Nod2* | Mouse | CAGGTCTCCGAGAGGGTACTG | GCTACGGATGAGCCAAATGAAG |
| *Tlr2* | Mouse | GCAAACGCTGTTCTGCTCAG | AGGCGTCTCCCTCTATTGTATT |
| *H2-Q7* | Mouse | GAGCAGGCTGGTATTGCAGAG | CACCATAAGACCTGGGGTGA |
| *Cd209f* | Mouse | CTCTTTGGGCCTCTTTTTGCT | AGTATGCACGAATCCTGGAGA |
| *Cd209g* | Mouse | GGCCTCAGCGATCACAGAAG | ACAACGGCTGTCATTCCATTTA |
| *Gapdh* | Mouse | CACCATGGGAGAAGGCCGGGG | GACGGACACATTGGGGGTAG |
| *C3* | Human | GGGGAGTCCCATGTACTCTATC | GGAAGTCGTGGACAGTAACAG |
| *THBS1* | Human | AGACTCCGCATCGCAAAGG | TCACCACGTTGTTGTCAAGGG |
| *CLEC4E* | Human | CTGAAACACAATGCACAGAGAGA | AAAGATGCGAAATGTCACAACAC |
| *CDC42EP2* | Human | TCCACCAAGGTGCCCATCTAT | TCCACCGATAACCGGGAGG |
| *CDC42EP4* | Human | GCGAGTCCTTGGACGAACAG | GCAGGGACATGGCATTCTTG |
| *OLR1* | Human | TTGCCTGGGATTAGTAGTGACC | GCTTGCTCTTGTGTTAGGAGGT |
| *NOD2* | Human | TGGTTCAGCCTCTCACGATGA | CAGGACACTCTCGAAGCCTT |
| *STX11* | Human | ACTTGTCCAAGCAATATGACCAG | CGTCTCGAACACGATGTCCTC |
| *TLR2* | Human | ATCCTCCAATCAGGCTTCTCT | GGACAGGTCAAGGCTTTTTACA |
| *GAPDH* | Human | GGAGCGAGATCCCTCCAAAAT | GGCTGTTGTCATACTTCTCATGG |
| *OVA* | Chicken | AGGGCTGACCATCCATTCCT | TTGTAGTCGGATCCAGGGGA |
